# Alternative Splicing of RNA Triplets Is Often Regulated and Accelerates Proteome Evolution

**DOI:** 10.1371/journal.pbio.1001229

**Published:** 2012-01-03

**Authors:** Robert K. Bradley, Jason Merkin, Nicole J. Lambert, Christopher B. Burge

**Affiliations:** Department of Biology, Massachusetts Institute of Technology, Cambridge, Massachusetts, United States of America; University of Bath, United Kingdom

## Abstract

Thousands of human genes contain introns ending in NAGNAG (N any nucleotide), where both NAGs can function as 3′ splice sites, yielding isoforms that differ by inclusion/exclusion of three bases. However, few models exist for how such splicing might be regulated, and some studies have concluded that NAGNAG splicing is purely stochastic and nonfunctional. Here, we used deep RNA-Seq data from 16 human and eight mouse tissues to analyze the regulation and evolution of NAGNAG splicing. Using both biological and technical replicates to estimate false discovery rates, we estimate that at least 25% of alternatively spliced NAGNAGs undergo tissue-specific regulation in mammals, and alternative splicing of strongly tissue-specific NAGNAGs was 10 times as likely to be conserved between species as was splicing of non-tissue-specific events, implying selective maintenance. Preferential use of the distal NAG was associated with distinct sequence features, including a more distal location of the branch point and presence of a pyrimidine immediately before the first NAG, and alteration of these features in a splicing reporter shifted splicing away from the distal site. Strikingly, alignments of orthologous exons revealed a ∼15-fold increase in the frequency of three base pair gaps at 3′ splice sites relative to nearby exon positions in both mammals and in *Drosophila*. Alternative splicing of NAGNAGs in human was associated with dramatically increased frequency of exon length changes at orthologous exon boundaries in rodents, and a model involving point mutations that create, destroy, or alter NAGNAGs can explain both the increased frequency and biased codon composition of gained/lost sequence observed at the beginnings of exons. This study shows that NAGNAG alternative splicing generates widespread differences between the proteomes of mammalian tissues, and suggests that the evolutionary trajectories of mammalian proteins are strongly biased by the locations and phases of the introns that interrupt coding sequences.

## Abstract 2

Inclusion or exclusion of single codons at the splice acceptor site of mammalian genes is regulated in a tissue-specific manner, is strongly conserved, and is associated with local accelerated protein evolution.

## Author Summary

In order to translate a gene into protein, all of the non-coding regions (introns) need to be removed from the transcript and the coding regions (exons) stitched back together to make an mRNA. Most human genes are alternatively spliced, allowing the selection of different combinations of exons to produce multiple distinct mRNAs and proteins. Many types of alternative splicing are known to play crucial roles in biological processes including cell fate determination, tumor metabolism, and apoptosis. In this study, we investigated a form of alternative splicing in which competing adjacent 3′ splice sites (or splice acceptor sites) generate mRNAs differing by just an RNA triplet, the size of a single codon. This mode of alternative splicing, known as NAGNAG splicing, affects thousands of human genes and has been known for a decade, but its potential regulation, physiological importance, and conservation across species have been disputed. Using high-throughput sequencing of cDNA (“RNA-Seq”) from human and mouse tissues, we found that single-codon splicing often shows strong tissue specificity. Regulated NAGNAG alternative splice sites are selectively conserved between human and mouse genes, suggesting that they are important for organismal fitness. We identified features of the competing splice sites that influence NAGNAG splicing, and validated their effects in cultured cells. Furthermore, we found that this mode of splicing is associated with accelerated and highly biased protein evolution at exon boundaries. Taken together, our analyses demonstrate that the inclusion or exclusion of RNA triplets at exon boundaries can be effectively regulated by the splicing machinery, and highlight an unexpected connection between RNA processing and protein evolution.

## Introduction

The split structure of eukaryotic genes impacts gene expression and evolution in diverse ways. Most directly, the presence of introns enables multiple distinct mRNA and protein products to be produced from the same gene locus through alternative splicing, which is often regulated between tissues or developmental stages [1],[2]. Alternative inclusion or exclusion of exons—“exon skipping”—can generate protein isoforms with distinct subcellular localization, enzymatic activity or allosteric regulation, and differing, even opposing, biological function [3]–[5]. Splicing is often regulated by enhancer or silencer motifs in the pre-mRNA that are bound by splicing regulatory proteins that interact with each other or with the core splicing machinery to promote or inhibit splicing at nearby splice sites [6]. Such enhancer and silencer motifs are common throughout constitutive as well as alternative exons and their flanking introns [7]–[9]. In turn, the presence of splicing regulatory motifs in exons, and their higher frequency near splice junctions, impacts protein evolution. For example, the frequencies of single nucleotide polymorphisms (SNPs) and amino acid substitutions are both reduced near exon-exon junctions relative to the centers of exons as a result of selection on exonic splicing enhancer motifs [10],[11]. Thus, a gene's exon-intron structure and its evolution are intimately linked.

Alternative 3′ and 5′ splice site use, in which longer or shorter versions of an exon are included in the mRNA, are among the most common types of alternative splicing in mammals [1] and can generate protein isoforms with subtly or dramatically differing function. For example, production of the pro-apoptotic Bcl-xS or the anti-apoptotic Bcl-xL protein isoforms is controlled through regulated alternative splice site usage [12]. Binding of splicing regulatory factors between the alternative splice sites or immediately adjacent to one site or the other can shift splicing toward the (intron-) proximal or distal splice site [6],[13],[14], providing a means to confer cell type-specific regulation. The distance between the alternative splice sites can vary over a wide range, from hundreds of bases to as few as three bases in the case of NAGNAG alternative 3′ splice sites.

**Figure 1:**
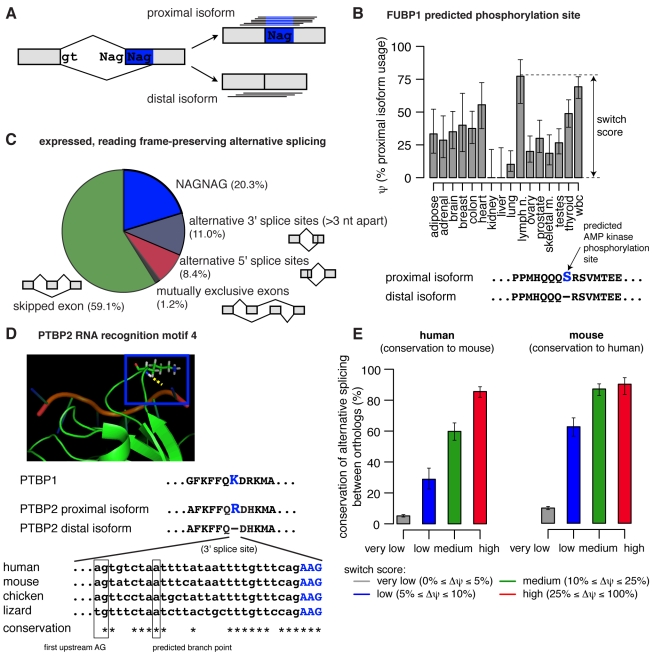
Alternative splicing of tissue-specific NAGNAGs is highly conserved. (A) Short reads were aligned to the intron-proximal and intron-distal splice junctions of NAGNAG splicing events in order to estimate isoform ratios. (B) Estimated proximal isoform usage (ψ) for a NAGNAG which inserts/deletes a predicted phosphorylation site in far upstream element binding protein 1 (FUBP1). Phosphorylation site and corresponding kinase were predicted by Scansite (Scansite *z*-score −3.024) [55]. Error bars indicate the 95% binomial confidence interval. (C) Number of reading frame-preserving alternative splicing events in protein-coding regions, with both isoforms expressed at ≥5% in at least one tissue (see also Table S1). (D) A NAGNAG which inserts/deletes an arginine in RNA recognition motif 4 (RRM4) of the splicing factor PTBP2 is deeply conserved. Alignment of orthologous 3′ splice site sequences shown below the NMR structure (PDB accession 2ADC, displayed with PyMOL) of the highly homologous PTBP1 protein (green) complexed with RNA (red) [33]. Boxed is K489 of PTBP1, which is homologous to the arginine shown in PTBP2, and hydrogen bonds to the RNA backbone (dotted yellow line). Putative branch point based on location of the first upstream AG, the sequence motif identified in [56], and the pattern of sequence conservation. (E) Conservation of alternative splicing between orthologous human and mouse NAGNAGs increases with tissue specificity. NAGNAGs that were alternatively spliced in human (left) and mouse (right) were grouped by switch score—defined as the maximum ψ difference between tissues—as indicated by colors, and the fraction of orthologs which were alternatively spliced in the other species is shown. Error bars indicate 95% binomial confidence intervals.

NAGNAG alternative splicing (Figure 1A) has been observed in vertebrates, insects, and plants, and is known to be very common. Bioinformatic analyses of expressed sequence tag (EST) databases have identified thousands of examples [15]–[18]. However, most of the mechanisms known to regulate other alternative 3′ splice site pairs, particularly those that involve binding of regulatory factors between the sites, or much closer to one site than the other, cannot apply to NAGNAGs because of the extreme proximity of the two sites. Thus, regulation of NAGNAGs is more difficult to envisage. Furthermore, analyses of select genes using PCR and capillary electrophoresis approaches reached differing conclusions about NAGNAG tissue specificity [15],[19],[20], and several authors have argued that NAGNAG splicing is purely stochastic, is not evolutionarily conserved, and is not physiologically relevant [21],[22]. However, analyses of NAGNAG splicing at a genome-wide scale have been hampered by the impracticality of distinguishing such similar isoforms by microarray hybridization and the insufficient depth of EST databases for assessment of tissue specificity.

In order to assess the abundance and potential regulation of NAGNAG splicing events genome-wide, we analyzed polyA-selected RNA-Seq data generated using the Illumina HiSeq platform from 16 human tissues at depths of ∼8 Gbp per tissue, similarly deep RNA-Seq data that we generated from eight mouse tissues, and data generated by the modENCODE consortium across a developmental time course in *Drosophila*. NAGNAG isoforms can be uniquely distinguished by short reads that overlap the splice junction, and the quantity of data available from each tissue in human and mouse typically represented at least 80-fold mean coverage of the transcriptome, a depth sufficient to detect potential tissue-specific differences in many cases. Sequence features were identified which can shift splicing toward the proximal or distal NAG, providing clues to regulation. We also analyzed the impact of NAGNAGs on exon evolution, obtaining evidence that NAGNAGs dramatically accelerate addition and deletion of sequence at the beginnings of exons.

## Results and Discussion

### Many Human NAGNAGs Are Regulated Across Tissues

Our initial analyses used the Illumina Body Map 2.0 dataset of polyA-selected RNA-Seq data from 16 human tissues (adipose, adrenal, brain, breast, colon, heart, kidney, liver, lung, lymph node, ovary, prostate, skeletal muscle, testes, thyroid, and white blood cells) sequenced at depths of ∼80 million paired-end 2×50 bp reads per tissue. This sequencing depth generates ∼8 Gbp of data, representing >80-fold coverage of the human protein-coding transcriptome. Enumerating all possible NAGNAG splicing events, we mapped both ends of each read against NAGNAG splice junctions (Figure 1A). Isoform ratios were estimated across all tissues as “percent spliced in” (PSI or ψ) values (Figure 1B), representing the fraction of mRNAs that use the intron-proximal splice site, thereby including the second NAG in the mRNA. The reliability of such RNA-Seq-based estimates of isoform abundance has been established previously [23].

Using a conservative approach that has comparable power to detect each of the major types of alternative splicing events, we estimated that NAGNAGs comprise slightly more than 20% of reading frame-preserving alternative splicing events in coding regions, making NAGNAGs the most common form of protein-producing alternative splicing after exon skipping (Figure 1C). In all, more than 2,000 NAGNAG events were detected in protein-coding regions of human genes where both isoforms were expressed at ≥5% in at least one tissue. Strikingly, 73% of these NAGNAGs showed evidence of tissue-specific regulation (*p*<0.01 by multinomial test). Furthermore, approximately 42% were “strongly regulated,” with changes in ψ of at least 25% between tissues (Table S1). For example, a NAGNAG in the gene encoding FUBP1, a transcriptional regulator of MYC, undergoes dramatically different splicing between kidney and lymph node (Figure 1B). Here, we report absolute rather than relative differences in splicing levels, e.g., a change from 10% to 35% between tissues is considered an increase of 25%, not 250%, and the largest difference in ψ between tissues is defined as the “switch score” [1]. Other genes containing NAGNAGs with switch scores of 50% or more included HOXD8, CAMK2B, ATRX, CAPRIN2, and MLLT4 (a complete list of human genes containing alternative NAGNAGs, sorted by switch score, is provided in Table S2). Technical replicates—sequencing of the same RNA-Seq libraries with 75 bp single-end reads at depths of ∼50 million reads per tissue—yielded similar estimates of NAGNAG abundance and regulation (Table S3).

### Regulated NAGNAGs Are Selectively Conserved between Primates and Rodents

Regulation that contributes to fitness is expected to be evolutionarily conserved. A previous study reported the existence of selection against alternatively spliced NAGNAGs in coding sequences [24]. Nevertheless, some NAGNAGs are quite deeply conserved, e.g., a NAGNAG that generates an arginine insertion/deletion in a RNA-binding domain of the splicing factor PTBP2 (also known as nPTB or brPTB). Both isoforms of this NAGNAG event are observed in ESTs from human, mouse, and chicken, and the potential for alternative splicing is conserved at the sequence level to lizard (Figure 1D). Consistent with this example, a previous analysis of EST databases suggested that a subset of alternatively spliced NAGNAGs are under purifying selection in vertebrates [25]. We systematically assessed the global conservation of NAGNAG isoform levels using RNA-Seq data generated from eight mouse tissues (brain, colon, kidney, liver, lung, skeletal muscle, spleen, and testes). Restricting to the set of NAGNAGs which were alternatively spliced in human (both isoforms expressed at ≥5% in at least one tissue), we found that NAGNAGs which were strongly regulated were approximately 10 times more likely than unregulated NAGNAGs to exhibit alternative splicing in their mouse orthologs, and vice versa (Figure 1E). This large and consistent increase in conservation of alternative splicing with increasing switch score suggests that regulated NAGNAGs are much more likely to contribute to organismal fitness, and therefore to be selectively maintained, than are alternatively spliced events which do not exhibit tissue specificity. If NAGNAG alternative splicing were selectively neutral, then we would not expect to see a correlation between the observed degree of tissue specificity in one species and conservation of alternative splicing in the other species.

**Figure 2:**
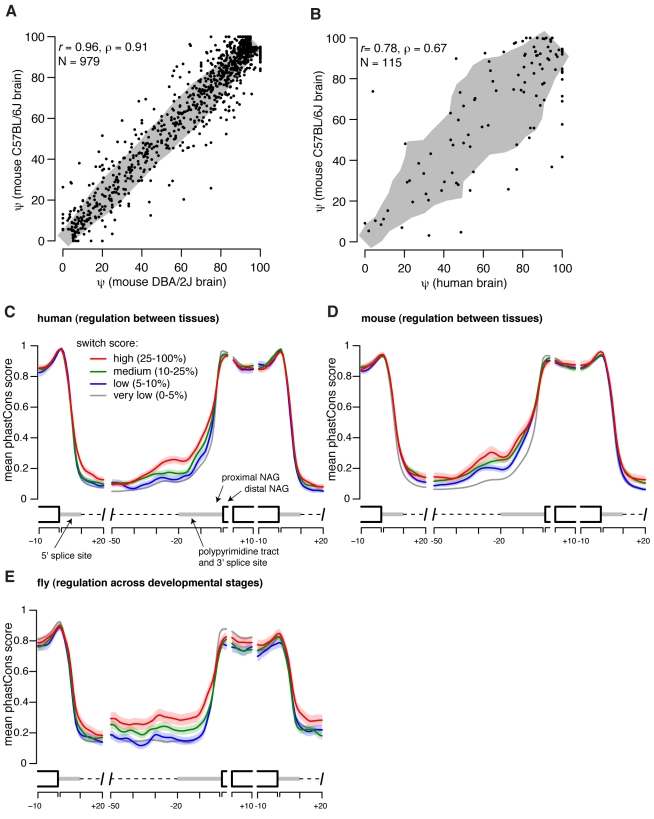
Increased sequence conservation upstream of tissue- and developmentally-regulated NAGNAGs. (A) NAGNAG ψ estimates are highly consistent in brain RNA-Seq data from the mouse strains DBA/2J and C57BL/6J. Only NAGNAGs with both isoforms expressed at ≥5% in either strain are shown. The 75^th^ percentile of the deviation from the line *y = x* is shown in gray. (B) NAGNAG ψ estimates are quantitatively conserved between human and mouse brain. Only NAGNAGs with both isoforms expressed at ≥5% in either species and satisfying |proximal 3′ splice site score – distal 3′ splice site score|≤0.5 bits are plotted (splice sites scored by MaxEnt model [36]). Deviation from *y = x* shown as in (A). (C) Sequence conservation of human NAGNAGs, where all NAGNAGs are aligned by their 3′ splice site junctions and grouped by switch score. Mean (solid line) and standard error of the mean (shaded area about solid line) of phastCons score [50] shown by position (averaged over a 2 nt sliding window) for each switch score category. Analysis restricted to human NAGNAGs for which the two AGs were conserved at the sequence level in mouse. (D) As in (C), but grouped by switch score in mouse and restricted to mouse NAGNAGs for which the two AGs were conserved at the sequence level in human. (E) As in (D), but for NAGNAGs in *Drosophila melanogaster*, with switch score defined across developmental stages rather than between tissues. Analysis restricted to *D. melanogaster* NAGNAGs for which the two AGs were conserved at the sequence level in *D. yakuba*.

NAGNAG isoform levels were very well correlated between biological replicates, consisting of individual mice of strains C57BL/6J and DBA/2J, whose genomes differ to an extent similar to that of unrelated humans (*r* = 0.96, Figure 2A), demonstrating the robustness and reproducibility of these RNA-Seq-based estimates of NAGNAG ψ values. Similar numbers of alternatively spliced NAGNAGs were detected in mouse as in human, with 28% of alternatively spliced NAGNAGs in mouse exhibiting evidence of tissue-specific regulation and 8% being strongly regulated across the eight tissues studied (Table S4). Many orthologous NAGNAGs in human and mouse exhibited tissue-specific regulation in both species, e.g., NAGNAGs in FUBP1, CAMK2B, CAPRIN2, and ATRX (a complete list of alternative NAGNAGs in mouse is provided in Table S5). The higher fraction of regulated NAGNAGs detected in the human data probably results from a combination of factors, including the greater number of tissues sampled (Figure S1), the diverse genetic backgrounds of the human samples, and intrinsically higher read coverage variability in the human RNA-Seq data used. Comparing technical replicates of human tissues, which capture variability in sequencing, we estimated false discovery rates (FDRs) for discovering strongly regulated NAGNAGs ranging from ∼0.8% to ∼13.3%, with a mean FDR of 4.4% (Figure S2). In contrast, comparing biological replicates of mouse tissues, which capture all major sources of variability (tissue collection, library preparation, sequencing, and individual-specific splicing differences), we estimated FDRs ranging from 0.6% to 1.9%, with a mean of 1.1% (Figure S3). Using these estimated FDRs, and extrapolating the mouse data to 16 tissues (Figure S1), we estimated that between 12% and 37% of NAGNAGs are strongly regulated across tissues in mammals, making strong regulation a fairly common occurrence—though somewhat less common than for other types of splicing events. The relatively small differences between samples of the same tissue from mice whose genomes differed to an extent comparable to that of unrelated humans (Figure 2A) suggested that inter-individual variation contributed less than other sources of variation (e.g., tissue-specific differences) to the variations observed between the human libraries.

Orthologous human and mouse NAGNAGs exhibited high quantitative conservation of isoform levels. This was particularly true when the difference between the proximal and distal 3′ splice site scores—using a method that scores the strength of the polypyrimidine tract and AG region—was conserved (Spearman's ρ = 0.67, Figure 2B). The correlation decreased somewhat in cases where the differences in 3′ splice site scores were less conserved (ρ = 0.54, *p* = 0.013 for test of equality of correlation using the Fisher transformation; Figure S4), suggesting that changes in relative 3′ splice site strength may contribute to species-specific differences in NAGNAG splicing. Notably, many NAGNAGs with diverged splice site scores were alternatively spliced in one species but constitutively spliced in the other, suggesting relatively rapid evolution of 3′ splice site positions.

### Regulated NAGNAGs Have Conserved Upstream Intronic Sequence

To better understand how NAGNAG splicing is regulated, and which sequence regions might be involved, we examined sequence conservation of flanking intronic and exonic regions for NAGNAGs grouped by switch score using alignments of the genomes of placental mammals. Tissue-specific NAGNAGs exhibited markedly increased sequence conservation in the upstream intron (Figure 2C–D), with little or no increase in other analyzed regions. The consistent increase in conservation in the upstream intron with increasing switch score provides further evidence that these regulated NAGNAGs contribute to organismal fitness, and is consistent with previous observations that alternatively spliced NAGNAGs have higher upstream sequence conservation than constitutive 3′ splice sites [26]. Enumerating NAGNAGs in introns of the fly *Drosophila melanogaster*, and comparing isoform usage across 30 developmental time points (embryo to adult) using RNA-Seq data from the modENCODE consortium [2], we identified over 500 NAGNAGs in coding regions of *Drosophila* genes where both isoforms were expressed at ≥5% in at least one developmental time point. Of these, 14% were developmentally regulated, with 5% being strongly regulated as defined above. As in mammals, more highly regulated fly NAGNAGs were associated with increased sequence conservation within and upstream of the 3′ splice site (Figure 2E). The consistent location of the sequence conservation signal for regulated NAGNAGs in mammalian and insect genomes (Figure 2C–E) suggested that the region ∼50 bp upstream of the NAGNAG motif, encompassing the competing 3′ splice sites themselves, may contain most of the regulatory information that governs NAGNAG alternative splicing. The extensive tissue-specific regulation observed in mammals and developmental regulation seen in flies may indicate that regulated NAGNAG alternative splicing is widespread in metazoans.

### Splice Site Score Difference Explains Mean NAGNAG Isoform Expression

**Figure 3:**
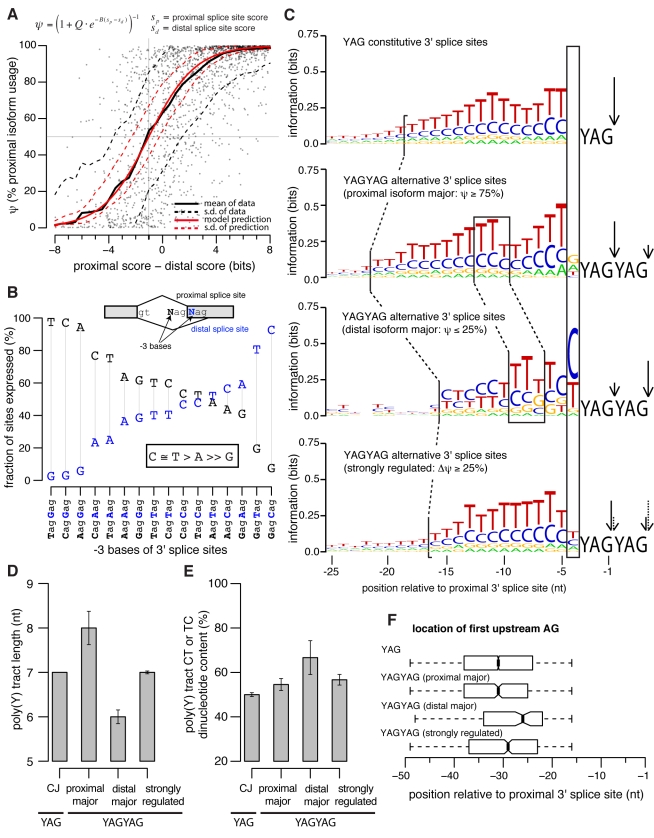
Variation in 3′ splice site features are associated with differences in NAGNAG splicing. (A) A simple biophysical model of NAGNAG splicing accurately models mean isoform usage across tissues as a function of difference in 3′ splice site score. Each point represents a single human NAGNAG, and the solid and dashed black lines show the mean ψ (across values for individual NAGNAGs with similar splice site score difference, with sliding window of 3.25 bits) and the standard deviation about the mean. The solid red line shows the prediction based on the model for parameters Q = 0.55 and B = 0.58, and the dashed red line indicates the standard deviation about the model mean expected from measurement error. The horizontal and vertical dashed lines indicate the splice site score difference (approximately 1 bit) at ψ = 50%. (B) The −3 bases largely determine whether a NAGNAG is alternatively spliced. We grouped NAGNAGs in the human genome according to their −3 bases and computed the fraction of each group which expressed the proximal (black) or distal (blue) isoform at ≥5% in at least one tissue. (C) Constitutive 3′ splice sites (top, YAG), YAGYAGs which express the proximal isoform at ≥75% in all tissues (middle, YAGYAG proximal major), YAGYAGs which express the distal isoform at ≥75% in all tissues (middle, YAGYAG distal major), and strongly regulated YAGYAGs (bottom, YAGYAG strongly regulated) all exhibit distinct upstream sequence preferences. The *x*-axis shows the position relative to the 3′ splice site (YAG) or proximal 3′ splice site (YAGYAG), and arrows indicate the 3′ splice site that is predominantly used. Figure was created with WebLogo [53]. Human and mouse YAGYAGs were grouped together to increase the statistical signal for (C–F). (D) Distal major YAGYAGs have shorter polypyrimidine tracts (*p*<0.001 relative to proximal major class, Kolmogorov-Smirnov test). Plot shows median length of the polypyrimidine tract, estimated as the first stretch of ≥5 consecutive pyrimidines upstream of the −3 position. Error bars indicate the standard deviation of the median, estimated by bootstrapping (the error bars for “CJ” were too small to be visible). (E) Distal major YAGYAGs have higher CT and TC dinucleotide content (*p*<0.005 relative to proximal major class, Kolmogorov-Smirnov test). Median CT and TC dinucleotide content of the polypyrimidine tract, computed as the fraction of the polypyrimidine tract composed of CT dinucleotides, with an optional T at the beginning or C at the end. Error bars indicate the standard deviation of the median, estimated by bootstrapping. (F) The AG exclusion zone [57] is more distally located in distal major YAGYAGs (*p*<0.001 relative to proximal major class, Kolmogorov-Smirnov test). Position of the first AG dinucleotide upstream of the −15 position is shown. Thick bars indicate the median positions, and boxes extend from the first to third quartiles.

The increased divergence in isoform usage observed for NAGNAGs that had undergone divergence in 3′ splice site score difference (Figures 2B, S4) suggested that relative splice site strength is a major determinant of NAGNAG quantitative isoform usage. Supporting this hypothesis, previous EST-based analyses have demonstrated that splice site strength impacts whether or not a NAGNAG will be alternatively spliced [21],[27]. To explore the relationship between splice site strength and quantitative isoform levels, rather than simply the presence or absence of alternative splicing, we created a biophysical model wherein the probabilities of using the proximal and distal splice sites are proportional to 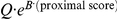 and 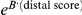, respectively, where the parameter 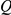 determines the inherent preference for using the intron-proximal splice site and 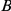 is a scaling factor for the splice site scores. This simple model, containing just two free parameters, accurately predicted mean isoform usage across human tissues (Figure 3A), suggesting that relative 3′ splice site strength is the primary determinant of basal NAGNAG isoform levels. The fitted value 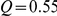 provides a quantitative measurement of preference for the proximal splice site in NAGNAG 3′ splice site recognition, predicting that the distal splice site of a NAGNAG must typically be 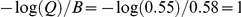 bit stronger than the proximal splice site in order to be spliced with equal efficiency. Analysis of mouse NAGNAGs yielded similar values of the Q and B parameters (Figure S5), supporting the robustness of these estimates. This preference for the proximal site was obvious even after controlling for the identity of the −3 bases (the Ns of the NAGNAG) (Figure 3B), which are known to be important determinants of NAGNAG isoform choice [18],[26],[27]. Preference for the proximal splice site is consistent with models of 3′ splice site recognition that involve scanning or diffusion from an upstream branch point [28],[29].

While the mean ψ value was accurately predicted by our model, the variability around the mean was substantially higher than expected based on measurement noise (Figure 3A). This observation is consistent with the concept that splice site strength determines the basal levels of the two NAGNAG isoforms, but the presence of regulatory sequence elements not captured by the 3′ splice site score, and variation in the levels of associated *trans*-acting factors, modulates the isoform ratios that occur in different tissues.

### Specific Sequence Features Associated with Basal and Regulated NAGNAG Splicing

The variability in NAGNAG splicing observed above implies that features outside of splice site strength and the −3 base must also be involved in determining isoform usage. For example, the NAGNAG in the splicing factor PTBP2 (Figure 1D) represents an exception to the pattern observed above: the −3 bases (CAGAAG) predict predominant proximal splice site usage, since C is strongly favored over A and is also proximal, but roughly equal proportions of both isoforms are expressed across all tissues studied (Figure S6). This observation led us to wonder whether other aspects of this 3′ splice site, e.g., the relatively short and distally located polypyrimidine tract and the relatively distal location of the putative branch point (Figure 1D) might favor use of the distal NAG in this and other cases.

While many analyses support the importance of the −3 base combination in NAGNAG alternative splicing [18],[26],[27], there is less consensus in the literature about the relevance of other major elements of the 3′ splice site, including the polypyrimidine tract and branch site. Molecular genetics experiments demonstrated that mutating sequences near the polypyrimidine tract and branch site influenced alternative splicing of specific NAGNAGs [30],[31], but two computational studies that used machine-learning approaches [27],[32] concluded that neither of these elements significantly influenced NAGNAG splicing globally. Notably, the experimental studies [30],[31] measured quantitative isoform ratios, as we do in this study, while the machine-learning studies [27],[32] simply classified NAGNAGs as constitutively or alternatively spliced.

In order to dissect features that impact NAGNAG isoform choice, controlling for the effect of the −3 bases, we considered the large class of NAGNAGs with favored (C or T) nucleotides at both −3 bases (YAGYAGs). We found that exons that predominantly used the proximal splice site (“proximal major” YAGYAGs) had substantially distinct nucleotide preferences from those that predominantly used the distal site (“distal major” YAGYAGs) (Figure 3C), consistent with the experimental results of Tsai et al. [30],[31], who found that modifying the sequence upstream of the 3′ splice site influenced NAGNAG splicing. For example, distal major YAGYAGs tended to have shorter, more distal, polypyrimidine tracts than proximal major YAGYAGs (Figure 3D), implicating polypyrimidine tract length and location in control of NAGNAG splicing. The proportion of CT/TC dinucleotides in the polypyrimidine tract was ∼25% higher for distal major YAGYAGs (Figure 3E), suggesting the possible involvement of CU/UC-binding factors such as those of the PTB family [33]—some of which are tissue-specifically expressed—in promoting use of distal NAGs. The location of the first upstream AG was also shifted several bases downstream in distal major YAGYAGs compared to other 3′ splice sites (Figure 3F), suggesting that the branch site is located further downstream in this class and that use of a distally located branch site favors use of the distal YAG, perhaps because the distance to the 3′ splice site is more optimal.

Strongly regulated YAGYAGs had features that were intermediate between the extremes found for proximal major and distal major YAGYAGs, such as polypyrimidine tracts of intermediate length (Figure 3D), suggesting that the presence of intermediate features facilitates regulation. Increased regulation was also associated with reduced 3′ splice site strength and greater similarity in strength between the competing sites (Figure S7), consistent with previous studies of other types of alternative splicing [34].

The −4 base, four nucleotides upstream of the 3′ splice site, is not generally considered to be important in splicing (with rare exceptions [35]). This position contains little or no information in alignments of constitutive 3′ splice sites [36], although a previous machine-learning analysis of features distinguishing between constitutively and alternatively spliced NAGNAGs included the −4 base in their classifier [27]. Our quantitative analysis strongly supported a special role in NAGNAG regulation for this canonically unimportant position. For distal major YAGYAGs, the −4 position (here referring to the position four nucleotides upstream of the intron-proximal splice site) had the highest information content of any position upstream of the YAGYAG (Figure 3C); furthermore, the −4 base was more conserved in distal major and strongly regulated YAGYAGs than for other classes of 3′ splice sites (Figure S8).

**Figure 4:**
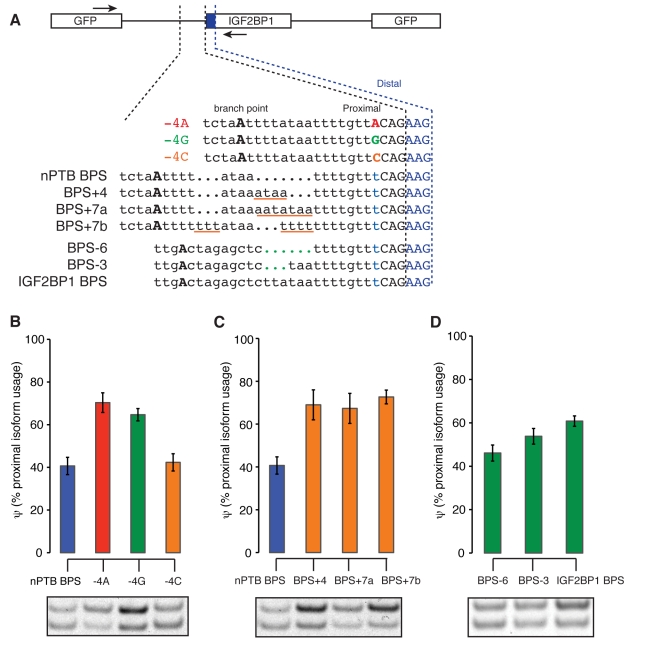
Specific intronic sequence features regulate NAGNAG splicing. (A) Illustration of NAGNAG minigene constructs, designed to test the roles of the branch point to 3′ splice site distance and of the −4 base in NAGNAG splicing. A short segment of intronic sequence spanning the branch point to the 3′ splice site of the PTBP2 NAGNAG was cloned upstream of the IGF2BP1 exon. To confirm the importance of a pyrimidine at the −4 position for distal NAG use, the effects of all four nucleotides at the −4 position were tested. The branch point to 3′ splice site distance was varied by introducing nucleotides (underlined in orange) in constructs containing the PTBP2 branch point sequence, or by removing nucleotides (indicated by green dots) in constructs containing the IGF2BP1 branch point sequence. Locations of RT-PCR primers are indicated by arrows. (B) Proximal isoform expression increased dramatically after the introduction of a purine at the −4 position. Splicing was monitored after minigene transfection into HEK293T cells by RT-PCR. Mean and standard deviation of at least three independent transfections are shown. A representative gel is shown below (top and bottom bands represent proximal and distal isoforms, respectively). (C) As in (B), but varying the branch point to 3′ splice site distance in the context of the native nPTB branch point sequence. The distance was increased by insertion of four or seven nucleotides of sequence of varying purine/pyrimidine composition as shown in (A). (D) As in (C), but decreasing the branch point to 3′ splice site distance in the context of the exogenous IGF2BP1 BPS by deletion of three or six bases as shown in (A).

Of the observations in Figure 3, the two that seemed most compelling were the preference for pyrimidines at the −4 position and the more distal positioning of branch points in YAGYAGs that favored the distal splice site. To test the predicted role of the −4 base in regulation of NAGNAG splicing, we used a minigene reporter based on the NAGNAG in PTBP2, whose splicing alters an exon coding for the RRM4 RNA binding domain (Figures 1D, 4A). As predicted based on the data in Figure 3C, mutation of the −4 base (T in the wildtype) to A or G resulted in a substantial shift in splicing toward use of the proximal NAG, while mutation to C had no effect (Figure 4B). These observations confirm that presence of a pyrimidine at the −4 position favors use of the distal NAG, even though no sequence preference was observed at this position in constitutive splice sites (Figure 3C). Presence of a pyrimidine at the −4 position of a NAGNAG might function to shift the location of binding of U2AF65 downstream by a base or more from its normal position, which might then result in preferential binding of U2AF35 to the downstream NAG, though this will require further study.

We also tested the role of the branch point in NAGNAG splicing by manipulating the branch site to 3′ splice site distance in this reporter, either in a context in which the inferred native branch point sequence (BPS) was intact or in a context in which the native BPS had been replaced by the previously mapped BPS of IGF2BP1 intron 11 (Figure 4A). With the native BPS present, an increase of just four bases in the BPS-3′ splice site distance was sufficient to cause a substantial shift in splicing towards the proximal NAG, with little or no additional shift resulting from addition of three more bases (Figure 4C). In the context of the exogenous IGF2BP1 BPS, a somewhat higher basal level of proximal splice site usage was reduced by deletion of six bases, with deletion of three bases producing a modest change (Figure 4D). These data indicate that the BPS plays a significant role in NAGNAG splicing and confirm that shorter BPS-3′ splice site distances can shift splicing toward the distal NAG.

### NAGNAGs Accelerate Protein Evolution at Exon-Exon Boundaries

**Figure 5:**
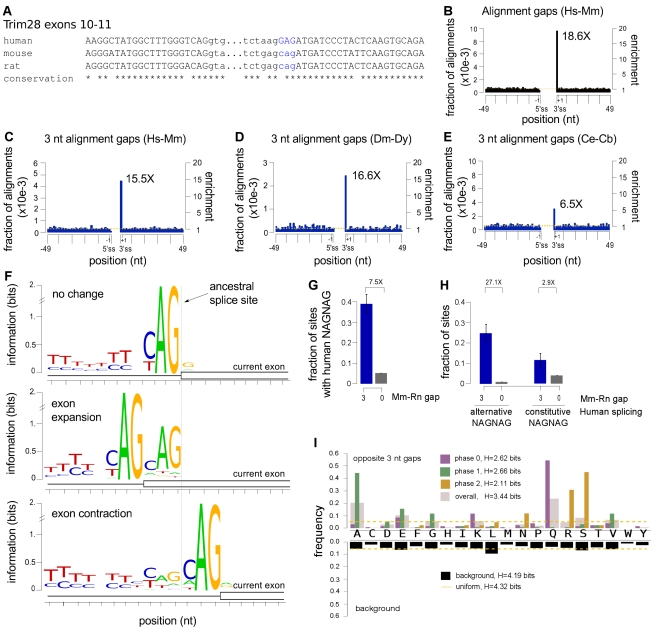
NAGNAGs are associated with accelerated protein evolution at exon-exon boundaries. (A) Alignment of portions of exons 10 and 11 of TRIM28 gene from three mammals, illustrating a shift in the upstream boundary of exon 11 between human and rodents. Exonic sequence shown in capitals; intronic sequence in lower case. (B) Gain/loss of exonic sequence between human and mouse occurs preferentially at 3′ splice sites (*p*<10^−6^, permutation test). The fraction of aligned orthologous human and mouse exons with gaps at each position is shown; the background level (mean fraction across the indicated region excluding the 3′ splice site) is shown by the dotted yellow line; the right-hand axis shows enrichment relative to this background. (C) As in (B), but restricted to gaps of length three bases. Preferential occurrence at 3′ splice sites was highly significant (*p*<10^−6^, permutation test). (D) Similar to (C), but based on alignments of orthologous *D. melanogaster* and *D. yakuba* exons. Preferential occurrence at 3′ splice sites was highly significant (*p*<10^−6^, permutation test). (E) Similar to (C), but based on alignments of orthologous *C. elegans* and *C. briggsae* exons. Preferential occurrence at 3′ splice sites was highly significant (*p*<10^−6^, permutation test). (F) Residual NAG motif at exons whose boundaries changed in the rodent lineage. Orthologous mouse and rat exons were classified as unchanged (top), expanded by three bases (middle), or contracted by three bases(bottom) based on comparison to an outgroup (human, cow, chicken, or *Xenopus laevis*), aligned to the inferred location of the ancestral 3′ splice site (dotted line). Information content of each position is shown relative to a uniform background composition. (G) Exons whose 3′ splice site boundaries differ by three bases between rat and mouse are 7.5 times as likely to have a NAGNAG in the human ortholog as exons whose boundaries did not change (*p*-value for difference<10^−24^ by Fisher's exact test). Error bars indicate the 95% binomial confidence interval. (H) Rodent exons orthologous to alternatively spliced human NAGNAG exons (left) are much more likely to exhibit exon boundary changes of three base pairs than those orthologous to constitutively spliced human NAGNAGs (right) (*p*-value for difference<10^−10^ by Fisher's exact test). Blue and gray bars in (H) represent subsets of blue and gray bars in (G), respectively. Error bars indicate the 95% binomial confidence interval. (I) Frequency of encoded amino acids that occur opposite gaps at the 3′ splice site in alignments of human and mouse exons is plotted above, overall (pink) and separately by the phase of the upstream intron (i.e., the number of bases, if any, in the last incomplete codon of the upstream exon); amino acid frequency at background positions (4 codons downstream of the 3′ splice site) is shown below. The Shannon entropy (a measure of randomness) of each amino acid frequency distribution is also shown.

Together, our analyses of proximal/distal major splicing suggested that NAGNAG 3′ splice sites afford broad scope for evolutionary tuning of isoform ratios, even in cases where the sequence of the second NAG is constrained by selection on the encoded amino acid. For example, mutations affecting the upstream −3 and −4 bases, the polypyrimidine tract, or the location of the branch site could all potentially modulate the ratio of the two isoforms across a range from predominantly proximal to predominantly distal isoform usage, which might facilitate evolutionary addition and deletion of single codons at 3′ splice junctions. A previous study observed reduced frequencies of amino acid substitutions near exon-exon junctions relative to the centers of exons, presumably resulting from purifying selection acting on exonic splicing enhancer motifs [10],[11]. By contrast, when we examined exon length changes in alignments of orthologous human and mouse coding exons (Figure 5A), we observed a striking 18.5-fold enrichment for gain/loss of exonic sequence at 3′ splice sites relative to flanking positions (Figure 5B; assignment of gaps is illustrated in example alignments in Figure S9). No particular enrichment for gain/loss of exonic sequence was observed at the 5′ splice site, suggesting that increased addition/deletion of exonic sequence is associated with properties of the 3′ splice site itself, rather than being a generic feature of exon boundaries. This pattern was not changed when restricting to constitutive splice junctions (Figure S10). A majority of the changes plotted in Figure 5B involved gain/loss of precisely three bases, and restricting to changes of exactly this size yielded a similar degree of enrichment at the 3′ splice site (Figure 5C).

While gain/loss of exonic sequence is normally attributed to insertions or deletions (“indels”) in the genome, the increased frequency of changes at the 3′ splice site suggested a prominent role for an alternative mechanism involving genomic substitutions that give rise to three base shifts in exon boundaries without insertion or deletion of genomic DNA. For example, creation of a NAG motif immediately upstream of a 3′ splice site NAG by mutation would be expected to commonly shift splicing upstream by three bases (resulting in exonization of three bases of intron) or generate an alternatively spliced NAGNAG that could subsequently lose splicing at the downstream NAG through mutation. Alternatively, a mutation creating an immediately downstream NAG—or a mutation that weakened the upstream NAG relative to a pre-existing downstream NAG—could result in either alternative splicing or loss of three bases of exonic sequence. As outlined in Table S6, both of these scenarios could arise frequently by single base substitutions, which occur at a rate that is an order of magnitude higher than the rate of genomic indels [37].

Consistent with this substitution/exaptation model and the finding that many NAGNAGs are alternatively spliced in the *Drosophila* lineage, we observed similar enrichment for gain/loss of three bases of exonic sequence at the 3′ splice site when comparing orthologous *D. melanogaster* and *D. yakuba* coding exons (Figure 5D). Notably, the enrichment of three base gaps at the 3′ splice site was 3-fold weaker in comparisons of *Caenorhabditis elegans* and *C. briggsae* exons (Figure 5E). NAGNAG alternative splicing is reported to occur rarely in nematodes due to a highly constrained 3′ splice site motif [15]. We confirmed the rarity of NAGNAG alternative splicing in *C. elegans* using RNA-Seq data from 14 developmental time points and conditions generated by the modENCODE consortium. Enumerating NAGNAGs in introns of *C. elegans* coding genes, we detected alternative splicing (both isoforms expressed at ≥5% in at least one developmental time point) for only 18% of NAGNAGs with favorable pyrimidine bases at both −3 positions based on RNA-Seq read depths slightly below those used in human. By contrast, 50%–85% of human, mouse, and *Drosophila* YAGYAGs were detected as alternatively spliced, suggesting that NAGNAG alternative splicing is substantially rarer in worms than in other metazoans. This decrease in abundance mirrors the 3-fold weaker enrichment of three base gaps at 3′ splice sites observed in worms (Figure 5E).

Sequence motif analyses further implicated NAGNAG splicing in the exon length changes observed at exon boundaries. Classifying the borders of orthologous mouse and rat exons as unchanged, expanded, or contracted (comparing to human, cow, chicken, and/or *Xenopus laevis* as outgroups), we observed evidence of residual NAGNAG motifs in exons with altered boundaries (Figure 5F). Specifically, exons expanded in mouse or rat exhibited a consensus NAG at exonic positions +1 to +3, and contracted exons exhibited a consensus NAG at intronic positions −6 to −4. The presence of this residual sequence motif provides further evidence that a substantial portion of exon length changes observed between orthologous mammalian exons derive from splicing-mediated shifts in exon boundaries rather than genomic indels. Likely because of subsequent selection to optimize the polypyrimidine tract, the residual NAG signal was weaker for contracted than for expanded exons.

Consistent with these findings, we observed a strong association between gain/loss of three bases in the rodent lineage and presence of a NAGNAG in orthologous human exons. Exons that expanded or contracted in rodents were 7.5-fold more likely to have a NAGNAG in the orthologous human exon than were exons with unchanged boundaries (Figure 5G). Further subdividing these exons according to the splicing pattern of the NAGNAG in human, we observed that rodent exons orthologous to alternatively spliced human NAGNAGs were ∼9 times more likely to have gained/lost exonic sequence than those orthologous to constitutively spliced human NAGNAGs (Figure 5H). These analyses implicate NAGNAG alternative splicing as a very common evolutionary intermediate in the gain and loss of single codons from exons.

This model, where frequent alternative splicing at the 3′ splice site leads to gain/loss of exonic sequence, is expected to play out very differently at 5′ splice sites. Competing 5′ splice sites are most frequently four bases apart [22], resulting in a frame-shift which is likely to render one of the protein products non-functional and potentially target the mRNA for nonsense-mediated decay. Although common, competing 5′ splice sites separated by four bases are therefore unlikely to lead to accelerated exon length changes and we observed no significant increase in exon length changes at the 5′ splice site (Figure 5A).

### NAGNAG-Accelerated Protein Evolution Is Highly Biased

Most three base changes to mRNAs probably minimally affect RNA-level properties such as message stability. However, insertion/deletion of a single amino acid residue can have a profound impact on protein function. For example, deletion of a single codon can alter protein degradation, subcellular localization, DNA binding affinity, or other protein properties [38],[39]; can cause diseases including cystic fibrosis and Tay-Sachs disease [40],[41]; and can even rescue a disease-related phenotype [42]. Insertion or deletion of a codon in a protein structural motif with a periodic hydrogen bonded structure such as a beta sheet or coiled coil domain might have a disproportionate effect on protein structure by altering the hydrogen bonding of a large number of downstream residues. The codon-level effects of NAGNAG splicing are largely determined by intron “phase” (position relative to the reading frame) [15]. Considering the spectrum of codons that occurred opposite three base gaps at the beginnings of exons (corresponding to the peak in Figure 5C), we observed a highly non-random distribution that strongly favored glutamine, alanine, glutamate, and serine and disfavored most other residues including cysteine, phenylalanine, and histidine relative to the background (Table S7). Distinct and far stronger biases were observed when grouping introns by phase. These biases occurred in a pattern consistent with frequent origin via exaptation of NAGNAGs (Figure 5I). For example, glutamine (mostly coded by CAG) was the most commonly added residue at the end of “phase 0” introns, for which the first three bases of the downstream exon form a codon. Serine (mostly AGY) and arginine (mostly AGR) were the most commonly added residues at the boundaries of phase 2 introns, for which the AG of an added NAG would form the first two bases of a codon. These biases contributed to a strong enrichment observed for gain/loss of predicted phosphorylation sites at 3′ splice sites (Figure S11). Together, the analyses in Figure 5 demonstrate that gain and loss of residues along proteins occurs in a strongly biased manner, with a highly accelerated rate and biased codon spectrum at the beginnings of exons that is likely driven by genomic substitutions that alter NAGNAG motifs or their splicing patterns. These observations suggest that the evolutionary trajectories of proteins in metazoans are shaped to a surprising extent by the specific locations and phases of introns that interrupt their coding sequences.

## Materials and Methods

### Accession Codes

Mapped sequence reads from the human and mouse RNA-Seq experiments are located in NCBI's GEO database (accession number GSE30017). The complete Body Map 2.0 sequence data are in the ENA archive with accession number ERP000546 (available at http://www.ebi.ac.uk/ena/data/view/ERP000546). These data are also accessible from ArrayExpress (ArrayExpress accession: E-MTAB-513). The Body Map 2.0 data were generated by the Expression Applications R&D group at Illumina using the standard (polyA-selected) Illumina RNA-Seq protocol from total RNA obtained commercially (Ambion) using the HiSeq 2000 system. We downloaded *D. melanogaster* (“Developmental Stage Timecourse Transcriptional Profiling with RNA-Seq”) and *C. elegans* (“Global Identification of Transcribed Regions of the C. elegans Genome”) RNA-Seq data from the modMINE (http://intermine.modencode.org/) website of the modENCODE consortium. For the *C. elegans* data, we restricted to 36 bp reads for consistency with other analyses.

### Splicing Events

We used the set of splicing events from [1] to identify skipped exons, alternative 3′ splice sites (>3 nt apart), alternative 5′ splice sites, and mutually exclusive exons in the human (GRCh37, or hg19) and mouse (NCBIM37, or mm9) genomes (Figure 1C). We enumerated all possible NAGNAGs in the human genome by finding all 3′ splice sites in these alternative splicing events and the Ensembl [43] and UCSC [44] annotation databases and then searching for NAGNAG motifs. We classified splice junctions as constitutive if they did not overlap any alternative splicing event present in the databases described above.

### Mouse Tissues and RNA-Seq Library Preparation

Mouse tissues from a 10-wk-old male were extracted immediately after death and stored in RNAlater per the manufacturer's instructions (Ambion). Tissue was lysed in Trizol and RNA was extracted with Qiagen miRNeasy mini columns. Using 5 µg of total RNA, we performed polyA selection and prepared strand-specific libraries for Illumina sequencing following the strand-specific dUTP protocol [45] and using the SPRIworks Fragment library system (Beckman Coulter). We obtained final insert sizes of approximately 160 bp. We sequenced these libraries using the Illumina HiSeq 2000 and the GAIIx machines.

### RNA-Seq Read Analysis

For each NAGNAG, we extracted the sequence flanking the proximal and distal 3′ splice sites and used Bowtie [46] version 0.12.7 to map reads to these two sequences. We required that short reads have at least 6 nt on either side of the splice junction (an “overhang” of 6 nt), and furthermore that there be no mismatches within the overhang region. In order to eliminate errors in read mapping due to non-unique splice junctions, we restricted the set of NAGNAGs enumerated across the genome to the subset of NAGNAGs for which all 36-mers mapping to either splice site did not map to the genome or any other splice junction (we used 36-mers because they were the shortest reads analyzed in our experiments). We then computed ψ values as (number of reads mapping to the proximal splice junction)/(number of reads mapping to either the proximal or distal splice junction). For all bioinformatics analyses, we only analyzed the subset of tissues for which a particular NAGNAG had a total of at least 10 reads in order to control for variation in junction coverage due to gene expression differences. We experimented with requiring different levels of junction coverage (10–100 reads per NAGNAG) and confirmed that our conclusions were insensitive to the chosen cutoff. We identified alternatively spliced events as those for which both isoforms were expressed at ≥5% in at least one sample (restricting to tissues for which a particular NAGNAG had ≥10 reads), and identified regulated events as those with *p*≤0.01 by the proportion or *z*-test (prop.test in R [http://www.R-project.org/]). As described in the text, when computing the fraction of regulated NAGNAGs, we only considered NAGNAGs which were alternative spliced by these criteria (both isoforms expressed at ≥5% in at least one sample).

For Figure 1C and Tables S2, S3, we re-mapped the reads using TopHat [47] version 1.1.4 and restricted to uniquely mapping reads with an overhang of 6 nt and no mismatches in the overhang region. Using only reads mapping to the two 3′ (skipped exons, NAGNAGs, alternative 3′ splice sites, and mutually exclusive exons) or 5′ (alternative 5′ splice sites) splice sites of each event, we computed ψ values and identified alternative spliced and regulated events as described above.

### False Discovery Rates

We estimated false-discovery rates as the fraction of events which were differentially expressed between technical (human) or biological (mouse) replicates identified using the procedure described above for regulated events. Briefly, for each tissue and pair of replicates, we restricted to the set of NAGNAGs which were alternatively spliced in at least one of the replicates and computed the fraction of these NAGNAGs which were differentially expressed with *p*≤0.01 between the replicates. We estimated mean FDRs for human (4.4%) and mouse (1.1%) by taking a weighted average over tissues, where we weighted the FDR computed for each tissue by the number of alternatively spliced NAGNAGs analyzed for that tissue.

The fraction of strongly regulated NAGNAGs increased essentially linearly with the number of tissues considered for both human and mouse (Figure S1). We expect this trend to continue as the number of mouse tissues increases, as it does for the human data. Accordingly extrapolating the mouse data to 16 tissues with a linear fit and subtracting the mean FDR of 1.1%, we estimated that at least 12% of alternatively spliced mouse NAGNAGs are strongly regulated, providing a lower bound on the fraction of strongly regulated NAGNAGs in mammals. We used the human data to compute a corresponding upper bound of 37% by subtracting the mean FDR of 4.4% from the observed fraction of strongly regulated NAGNAGs (Figure S1).

### Boltzmann Model

For each NAGNAG event, the probabilities of using the proximal and distal splice sites are proportional to 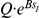 and 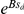, where 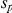 and 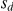 are the proximal and distal splice site scores. The probability of using the proximal splice site is therefore 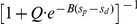. We fit the parameters 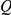 and 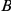 as follows: For each NAGNAG, we computed the mean ψ (averaging over tissues). We then binned NAGNAGs according to their splice site score differences, using a bin size of 3.25 bits and a bin increment of 0.5 bits, and computed the median ψ for each bin. We fit a straight line to the six bins flanking the point where ψ = 50% and estimated the parameters as 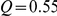 and 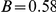 based on a first-order Taylor expansion.

### Ortholog Identification and Sequence Conservation Analysis

We performed a whole-genome alignment of human and mouse using Mercator (http://www.biostat.wisc.edu/~cdewey/mercator/) and FSA [48], and identified orthologous NAGNAGs as those for which both the 5′ splice site and competing 3′ splice sites were orthologous according to the corresponding sequence alignment. For the *Drosophila* analysis, we used a previously described *D. melanogaster*–*D. yakuba* whole-genome alignment [49].

For all sequence conservation analyses, we downloaded phastCons scores [50] from the UCSC annotation databases [44]. We used phastCons46 (placental mammals) for human, phastCons30way (placental mammals) for mouse, and phastConst15way for *D. melanogaster*.

### Minigene Assays

Segments of PTBP2 intronic sequence containing the NAGNAG were cloned into a modular splicing reporter [51] upstream of the IGF2BP1 exon using SacI and XhoI restriction enzyme sites. Forward and reverse oligonucleotides (below) were mixed in equimolar ratios, annealed, and double-digested with SacI and XhoI, or in some cases the oligonucleotides were ordered with desired restriction site overhangs, and ligated into the pGM4G9 minigene. For constructs analyzing the effects of distance to the native PTBP2 branch point, the vector (IGF2BP1) branch point sequence was first mutated by site-directed mutagenesis (TCATTGA was deleted, immediately upstream from the SacI restriction site) prior to insertion of the PTBP2 3′ splice site.

All minigene reporters (0.5 µg) were transfected into HEK293T cells using Lipofectamine 2000 (Invitrogen). RNA was isolated 18–24 h post-transfection with RNeasy Mini Kits (Qiagen). RT-PCR was performed with a fluorescent primer (NAGNAG_Forward: 5′ 6FAM- TCTTCAAGTCCGCCATGC and NAGNAG_reverse: 5′ AGTCAGGTGTTTCGGGTGGT). The proximal (63 nucleotides) and distal (60 nucleotides) isoforms were resolved on a 10% TBE gel and detected with a Typhoon 9000 scanner (GE Healthcare). Proximal and distal isoforms were quantified with ImageJ software.

Primers: PTB2_For: cagtgtctaattttataattttgtttcagAAGATTGCACCACCCGAAACACCTGACTCCAAAGTTCGTATGGTTC; PTB2_Rev: TCGAGAACCATACGAACTTTGGAGTCAGGTGTTTCGGGTGGTGCAATCTTctgaaacaaaattataaaattagacactgagct; BPS+4_For: cagtgtctaattttataaataattttgtttcagAAGATTGCACCACCCGAAACACCTGACTCCAAAGTTCGTATGGTTC; BPS+4_Rev: TCGAGAACCATACGAACTTTGGAGTCAGGTGTTTCGGGTGGTGCAATCTTctgaaacaaaattatttataaaattagacactgagct; BPS+7a_For: cagtgtctaattttataaataaatattttgtttcagAAGATTGCACCACCCGAAACACCTGACTCCAAAGTTCGTATGGTTC; BPS+7a_Rev: TCGAGAACCATACGAACTTTGGAGTCAGGTGTTTCGGGTGGTGCAATCTTctgaaacaaaatatttatttataaaattagacact gagct; BPS+7b_For: cagtgtctaatttttttataattttttttgtttcagAAGATTGCACCACCCGAAACACCTGACTCCAAAGTTCGTATGGTTC; BPS+7b_Rev: TCGAGAACCATACGAACTTTGGAGTCAGGTGTTTCGGGTGGTGCAATCTTctgaaacaaaaaaaattataaaaaaattagacactgagct; −4A_For: cagtgtctaattttataattttgttacagAAGATTGCACCACCCGAAACACCTGACTCCAAAGTTCGTATGGTTC; −4_Rev: TCGAGAACCATACGAACTTTGGAGTCAGGTGTTTCGGGTGGTGCAATCTTctgtaacaaaattataaaattagacactgagct; −4G_For: cagtgtctaattttataattttgttgcagAAGATTGCACCACCCGAAACACCTGACTCCAAAGTTCGTATGGTTc; −4G_Rev: TCGAGAACCATACGAACTTTGGAGTCAGGTGTTTCGGGTGGTGCAATCTTctgcaacaaaattataaaattagacactgagct; −4C_For: cagtgtctaattttataattttgttccagAAGATTGCACCACCCGAAACACCTGACTCCAAAGTTCGTATGGTTc; −4C_Rev: TCGAGAACCATACGAACTTTGGAGTCAGGTGTTTCGGGTGGTGCAATCTTctggaacaaaattataaaattagacactgagct; IGF2BP1BPS_For: gcgagctcttataattttgtttcagAAGATTGCACCACCCGAAACACCTGACTCCAAAGTTCGTATGGTTCTCGAGCGG; IGF2BP1BPS_Rev: CCGCTCGAGAACCATACGAACTTTGGAGTCAGGTGTTTCGGGTGGTGCAATCTTctgaaacaaaattataagagctcgc; BPS-3_For: gcgagctctaattttgtttcagAAGATTGCACCACCCGAAACACCTGACTCCAAAGTTCGTATGGTTCTCGAGCGG; BPS-3_Rev: CCGCTCGAGAACCATACGAACTTTGGAGTCAGGTGTTTCGGGTGGTGCAATCTTctgaaacaaaattagagctcgc; BPS-6_For: gcgagctcttttgtttcagAAGATTGCACCACCCGAAACACCTGACTCCAAAGTTCGTATGGTTCTCGAGCGG; BPS-6_Rev: CCGCTCGAGAACCATACGAACTTTGGAGTCAGGTGTTTCGGGTGGTGCAATCTTctgaaacaaaagagctcgc.

### Evolutionary Analysis

We restricted all analyses to “singleton orthologs,” genes without paralogs and with unambiguous orthology assignments in all species considered for each analysis, annotated in Ensembl [43] and queried with PyCogent [52]. For each gene, we required that the longest annotated coding sequence have the same number of exons in all species, and performed all subsequent analyses using this longest coding sequence. For each longest coding sequence, we extracted pairs of consecutive exons, concatenated them, and then aligned them to their corresponding orthologous sequences using FSA [48]. In order to control for alignment error, we required that alignment sequence identity be greater than 70% and that the total inserted sequence be no longer than 20% of the length of the shortest exon. Furthermore, if gaps in an alignment could be moved to lie at exon-exon boundaries rather than within exonic sequence while preserving the alignment quality (number of exact matches), then we modified the alignment accordingly, as FSA is unaware of exon structures. This modification affected only a small fraction of alignments, and our results in Figure 5 are unchanged without this modification.

We classified orthologous mouse and rat exons as unchanged, expanded, or contracted based on comparison with an outgroup (human, cow, chicken, *Xenopus laevis*, or *Danio rerio*, in that order, until an informative comparison was found). For each exon in each class, we extracted the corresponding intronic sequence and created a sequence logo using WebLogo (Figure 5F–H) [53].

For analyses of amino acid sequences in Figure 5I, we compared the amino acids gained or lost in alignments with gaps of three bases at the 3′ splice site. If the next gain/loss was a single amino acid (for example, if the human peptide was SR and the mouse peptide was R), then we counted only the single amino acid which was inserted (S); if the gain/loss was two amino acids (for example, if the human peptide was SR and the mouse peptide was K), then we counted both amino acids which were inserted (SR).

For Figure S11, we used a BioPerl module [54] to query Scansite [55] to predict phosphorylation sites (medium stringency) in the translated longest annotated coding sequence, and plotted the location of predicted phosphorylation sites which were gained/lost in human and mouse.

Unless otherwise described, all plots in Figure 5 were created with matplotlib (http://matplotlib.sourceforge.net/).

## Supporting Information

Figure S1 Dependence of the fraction of strongly regulated NAGNAGs on the number of tissues. (A) Human. (B) Mouse.
(TIFF)

Figure S2 Technical variability in human libraries. Single-end (75 bp) and paired-end (2×50 bp) sequencing of the same human libraries captures sequencing variability.
(TIFF)

Figure S3 Biological variability in mouse libraries. Sequencing of mouse libraries created from two different individuals captures all major sources of variability, including library preparation (2×36 bp versus 2×80 bp), sequencing, sample collection, and individual-specific splicing (C57BL/6J versus DBA/2J).
(TIFF)

Figure S4 Correlation between human and mouse isoform usage patterns for NAGNAGs with diverged differences in splice site scores. As Figure 2B, but for NAGNAGs with |proximal splice site score – distal splice site score|>0.5.
(TIFF)

Figure S5 Biophysical models of NAGNAG isoform usage in different species. (A) Human (identical to Figure 3A). (B) Mouse.
(TIFF)

Figure S6 Isoform usage of the NAGNAG in the PTBP2 gene illustrated in Figure 1D. (A) Human. (B) Mouse.
(TIFF)

Figure S7 Splice site score difference and maximum splice site score as a function of switch score for different classes of alternative 3′ splice sites. (A) The splice site scores of regulated NAGNAG 3′ splice sites tended to be far more similar to one another than those of unregulated events, suggesting that regulation is easier to achieve when the intrinsic strengths of the sites are evenly matched. (B–C) This trend was much weaker for more distant alternative 3′ splice site events. (D) The 3′ splice site scores of tissue-regulated NAGNAGs also tended to be somewhat weaker than for unregulated NAGNAGs or constitutive 3′ splice sites. This observation suggested that weaker splice sites are more easily regulated, consistent with previous studies of other types of alternative splicing. (E–F) This trend for regulated events to be associated with weaker splice site scores was observed to a much lesser extent for alternative 3′ splice sites separated by longer distances, suggesting that splicing regulatory elements may more readily exert differential effects on more widely spaced 3′ splice sites, making matching of splice site scores less critical for achieving regulation for this class than it is for NAGNAGs. For example, we have previously shown that most exonic splicing silencer (ESS) elements inhibit the intron-proximal site when situated between competing 3′ splice sites, an arrangement that requires separation of the competing sites by sufficient space to accommodate the ESS, and so does not apply to NAGNAGs. “v. low” indicates “very low,” and “CJ” indicates the 3′ splice sites of constitutive junctions.
(TIFF)

Figure S8 Relative conservation at the −4 position for different classes of NAGNAGs. Plot shows median relative conservation at the −4 position, computed as (phastCons score at −4 position/phastCons score at −3 position). “CJ” indicates the 3′ splice sites of constitutive junctions. Error bars indicate the standard error of the median, estimated by bootstrapping.
(TIFF)

Figure S9 Numbering of alignment gaps relative to the 5′ and 3′ splice sites. Examples shown in the figure illustrate the numbering system used for assessing gap positions relative to the 5′ and 3′ splice sites. The splice sites are numbered 0, and gap position is numbered relative to the nearest splice site. Gaps that could not be unambiguously assigned to one splice site were very rare and their inclusion or exclusion did not affect our conclusions.
(TIFF)

Figure S10 Exons with constitutively spliced NAGNAGs show an enrichment for gaps at the 3′ splice site. We restricted our analysis in Figure 5B to exons containing NAGNAGs which were constitutively spliced (ψ<5% or ψ>95% across all tissues) in both human and mouse. We observed qualitatively similar patterns of specific enrichment of gaps at the 3′ splice site, suggesting that the signal observed in Figure 5B was not due to unannotated alternative splicing of NAGNAGs.
(TIFF)

Figure S11 Alignment gaps at splice sites are enriched for predicted phosphorylation sites. The distribution of alignment gaps containing one or more predicted phosphorylation sites is shown for (A) all gaps and (B) gaps of three bases.
(TIFF)

Table S1 Abundance and regulation of alternative splicing events in human protein-coding sequence (paired-end sequencing). “No. of events” shows the abundance of alternative splicing events in protein-coding sequence, restricted to events for which (1) neither isoform is predicted to be targeted by nonsense-mediated decay (no splice junction ≥50 nt downstream of the stop codon), and (2) both isoforms are expressed at ≥5% in at least one tissue. Isoform ratios are based on the numbers of reads aligning to the 3′ splice sites of each isoform, thereby treating each event as a choice between competing 3′ splice sites (with the exception of alternative 5′ splice site events, where the reads aligning to 5′ splice sites were used). This method ensures that the different classes of splicing events are analyzed “fairly,” irrespective of the length of the alternatively spliced sequence. “Fraction strongly regulated” gives raw estimates (not corrected for using FDRs based on technical replicates).
(DOCX)

Table S2 Complete list of human genes containing alternatively spliced NAGNAGs, sorted by estimated switch score.
(XLS)

Table S3 Abundance and regulation of alternative splicing events in human protein-coding sequence (single-end sequencing). Identical to Table S1, but based on single-end, rather than paired-end, sequence data from Body Map 2.0.
(DOCX)

Table S4 Abundance and regulation of alternative splicing events in mouse protein-coding sequence. Similar to Table S1, but for mouse NAGNAGs and based on paired-end sequencing of a C57BL/6J individual.
(DOCX)

Table S5 Complete list of mouse genes containing alternatively spliced NAGNAGs, sorted by estimated switch score.
(XLS)

Table S6 Models for evolutionary gain and loss of single codons at 3′ splice sites resulting from splicing changes caused by single nucleotide substitutions. For each major category of change (bold headings in first column), the second column diagrams the effect on splicing, e.g., /… ⇒ …/ indicates a three base shift downstream in the location of the 3′ splice site, and …/ ⇒ /…/ indicates a change from constitutive splicing to alternative splicing at both the original 3′ splice site and a site three bases upstream. Below each diagram is a sequence motif consisting of specific bases (A, G), degenerate positions (N, indicating any base), or partially degenerate positions (B = “not A” = a C, G or T, H = “not G”, etc.). The third column lists conditions that are expected to favor each type of change (based on −3 base preferences shown in Figure 3B).
(DOCX)

Table S7 Number of amino acids gained or lost through 3′ splice site gaps are strongly biased depending on the intron phase. Table shows counts of amino acids gained/lost between orthologous human and mouse exons; the corresponding frequencies are shown in Figure 4I.
(DOCX)

## Abbreviations

BPS: branch point sequence
EST: expressed sequence tag
FDR: false discovery rate
PSI: percent spliced in
SNP: single nucleotide polymorphism
