# Supplementary figures and images for: Alternative Splicing of RNA Triplets Is Often Regulated and Accelerates Proteome Evolution
Source: PLoS Biol. 2012 Jan 3;10(1):e1001229. doi: 10.1371/journal.pbio.1001229 (PMC3250501; doi:10.1371/journal.pbio.1001229)

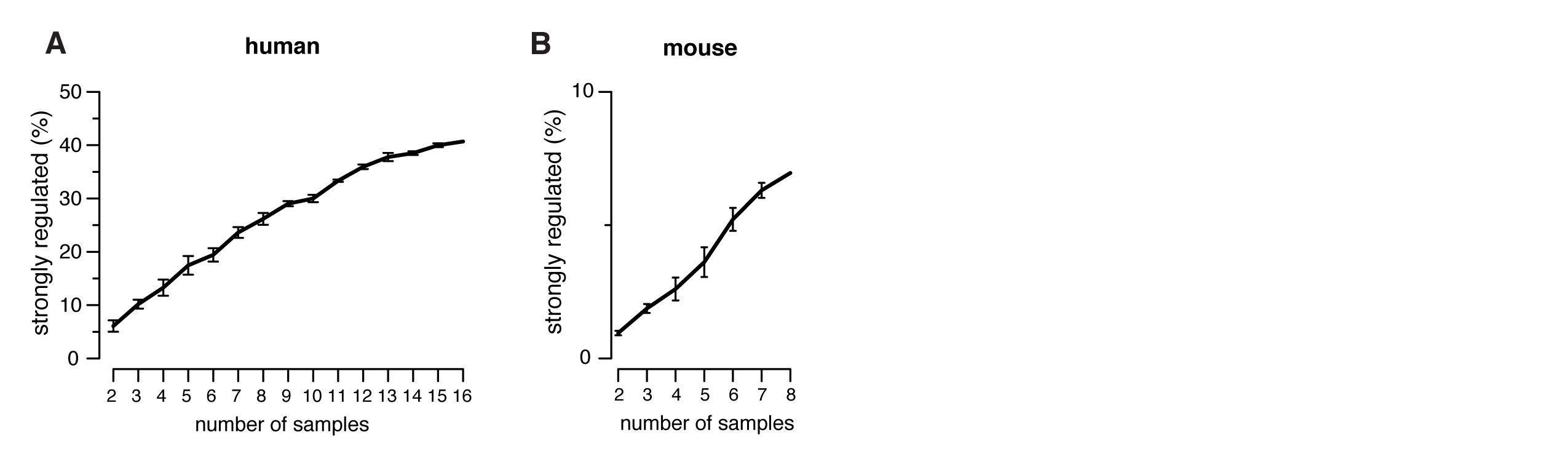

Supplement: Figure S1 — Dependence of the fraction of strongly regulated NAGNAGs on the number of tissues. (A) Human. (B) Mouse. (TIFF) [file pbio.1001229.s001.tiff]

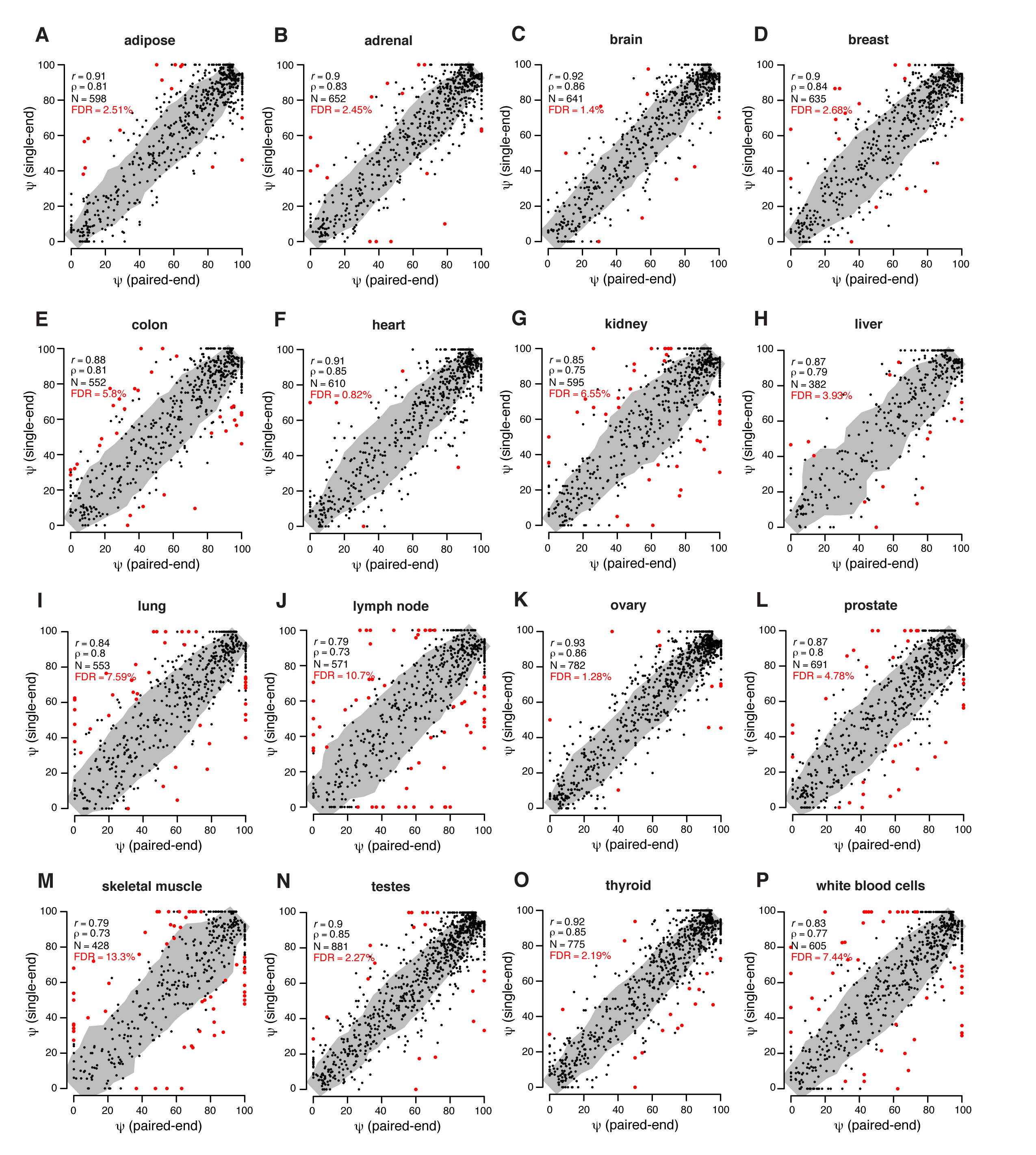

Supplement: Figure S2 — Technical variability in human libraries. Single-end (75 bp) and paired-end (2×50 bp) sequencing of the same human libraries captures sequencing variability. (TIFF) [file pbio.1001229.s002.tiff]

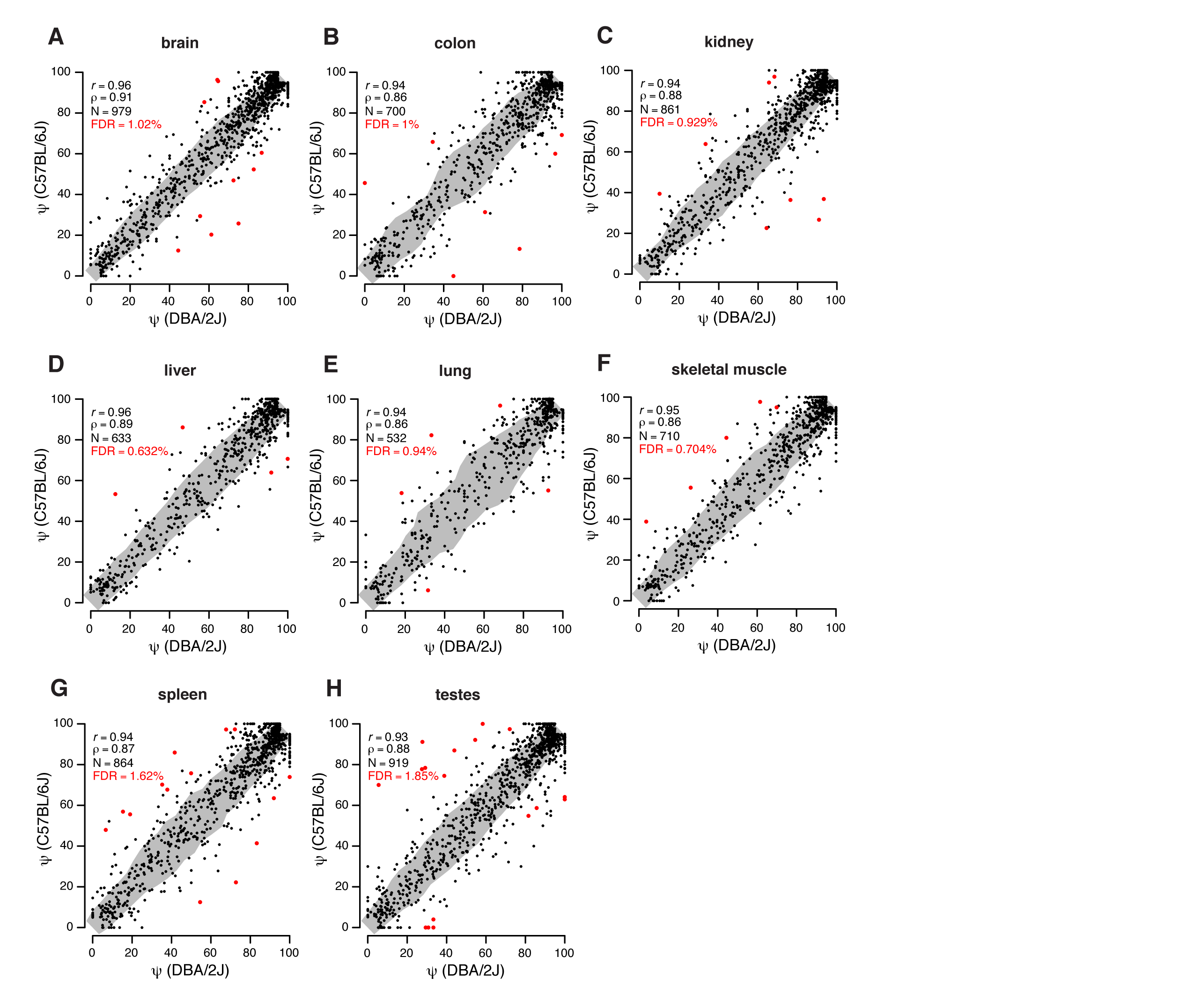

Supplement: Figure S3 — Biological variability in mouse libraries. Sequencing of mouse libraries created from two different individuals captures all major sources of variability, including library preparation (2×36 bp versus 2×80 bp), sequencing, sample collection, and individual-specific splicing (C57BL/6J versus DBA/2J). (TIFF) [file pbio.1001229.s003.tiff]

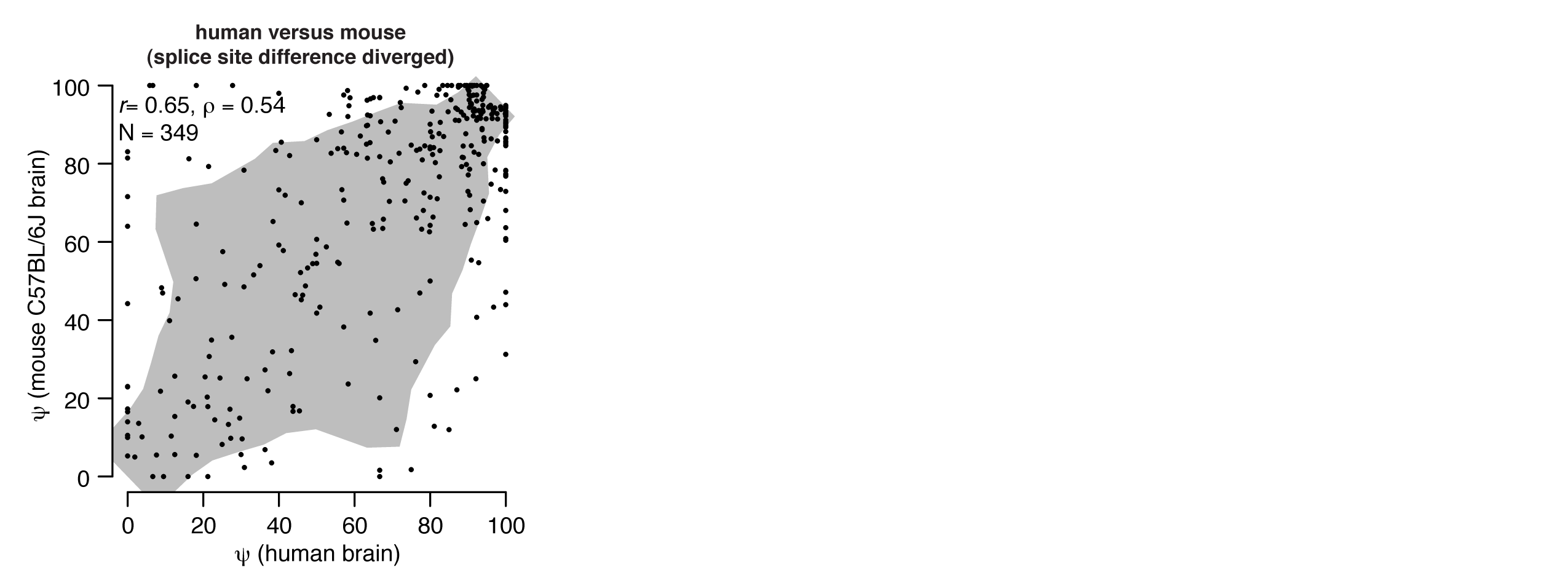

Supplement: Figure S4 — Correlation between human and mouse isoform usage patterns for NAGNAGs with diverged differences in splice site scores. As Figure 2B, but for NAGNAGs with |proximal splice site score – distal splice site score|>0.5. (TIFF) [file pbio.1001229.s004.tiff]

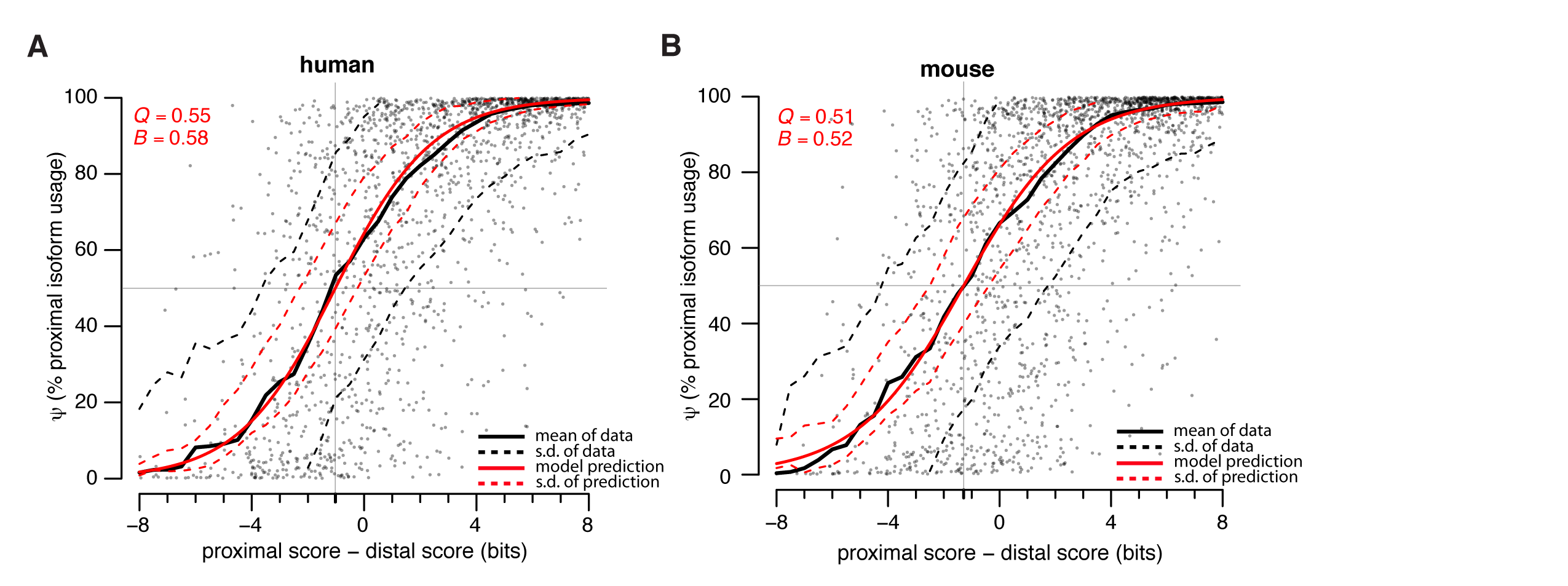

Supplement: Figure S5 — Biophysical models of NAGNAG isoform usage in different species. (A) Human (identical to Figure 3A). (B) Mouse. (TIFF) [file pbio.1001229.s005.tiff]

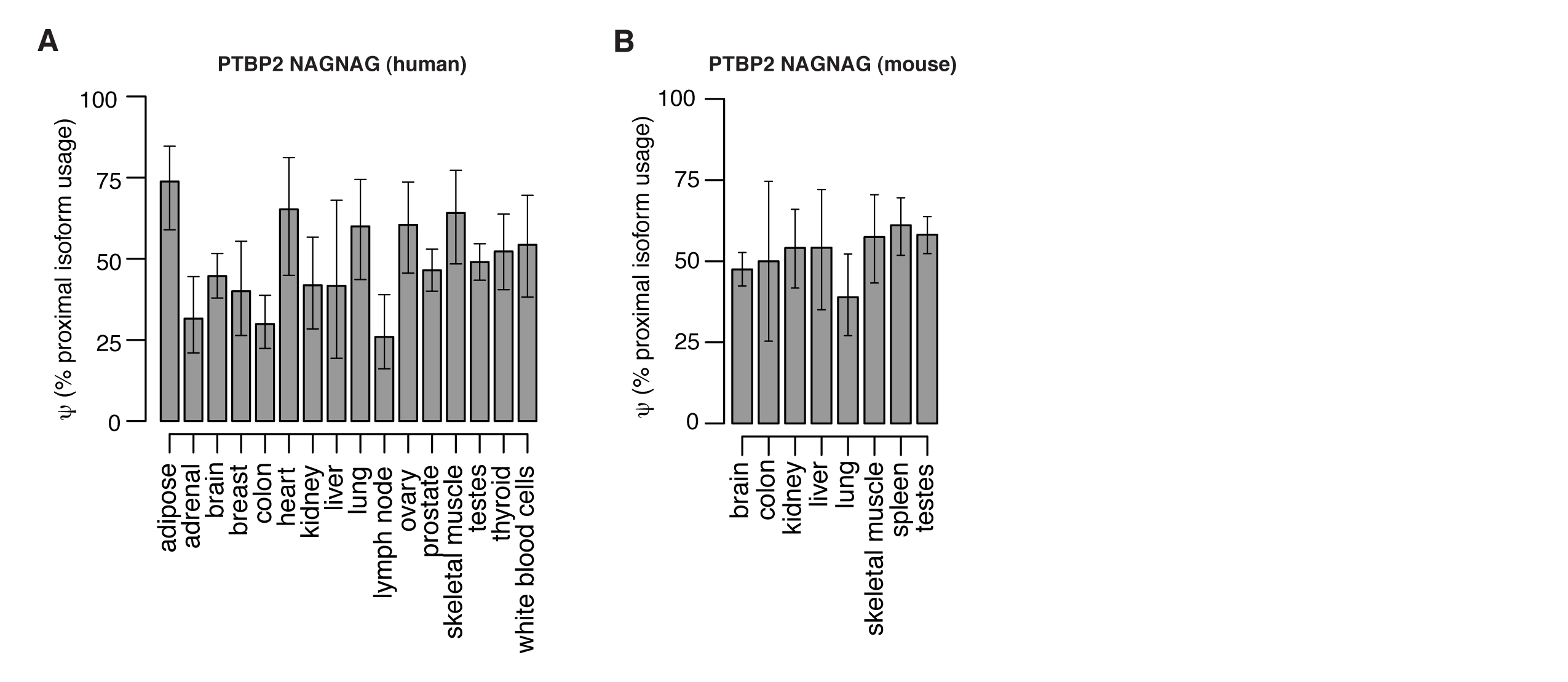

Supplement: Figure S6 — Isoform usage of the NAGNAG in the PTBP2 gene illustrated in Figure 1D. (A) Human. (B) Mouse. (TIFF) [file pbio.1001229.s006.tiff]

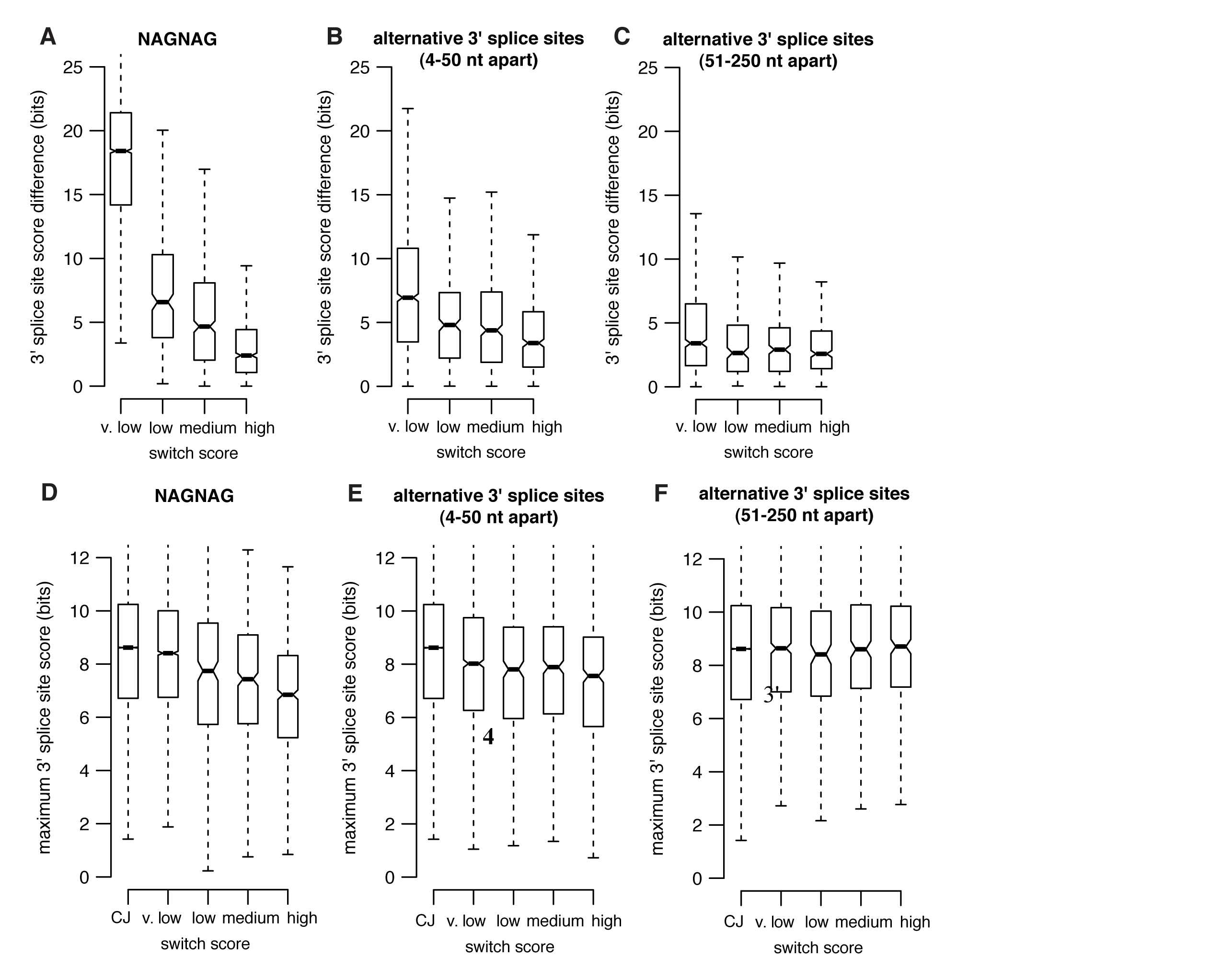

Supplement: Figure S7 — Splice site score difference and maximum splice site score as a function of switch score for different classes of alternative 3′ splice sites. (A) The splice site scores of regulated NAGNAG 3′ splice sites tended to be far more similar to one another than those of unregulated events, suggesting that regulation is easier to achieve when the intrinsic strengths of the sites are evenly matched. (B–C) This trend was much weaker for more distant alternative 3′ splice site events. (D) The 3′ splice site scores of tissue-regulated NAGNAGs also tended to be somewhat weaker than for unregulated NAGNAGs or constitutive 3′ splice sites. This observation suggested that weaker splice sites are more easily regulated, consistent with previous studies of other types of alternative splicing. (E–F) This trend for regulated events to be associated with weaker splice site scores was observed to a much lesser extent for alternative 3′ splice sites separated by longer distances, suggesting that splicing regulatory elements may more readily exert differential effects on more widely spaced 3′ splice sites, making matching of splice site scores less critical for achieving regulation for this class than it is for NAGNAGs. For example, we have previously shown that most exonic splicing silencer (ESS) elements inhibit the intron-proximal site when situated between competing 3′ splice sites, an arrangement that requires separation of the competing sites by sufficient space to accommodate the ESS, and so does not apply to NAGNAGs. “v. low” indicates “very low,” and “CJ” indicates the 3′ splice sites of constitutive junctions. (TIFF) [file pbio.1001229.s007.tiff]

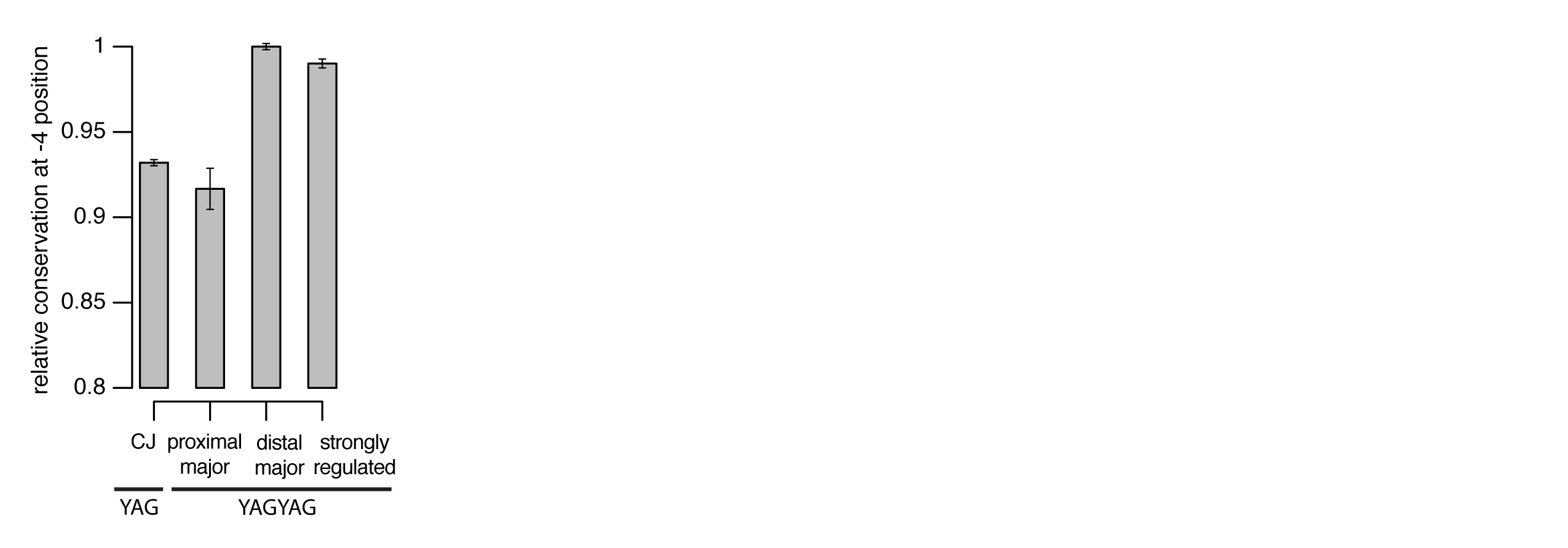

Supplement: Figure S8 — Relative conservation at the −4 position for different classes of NAGNAGs. Plot shows median relative conservation at the −4 position, computed as (phastCons score at −4 position/phastCons score at −3 position). “CJ” indicates the 3′ splice sites of constitutive junctions. Error bars indicate the standard error of the median, estimated by bootstrapping. (TIFF) [file pbio.1001229.s008.tiff]

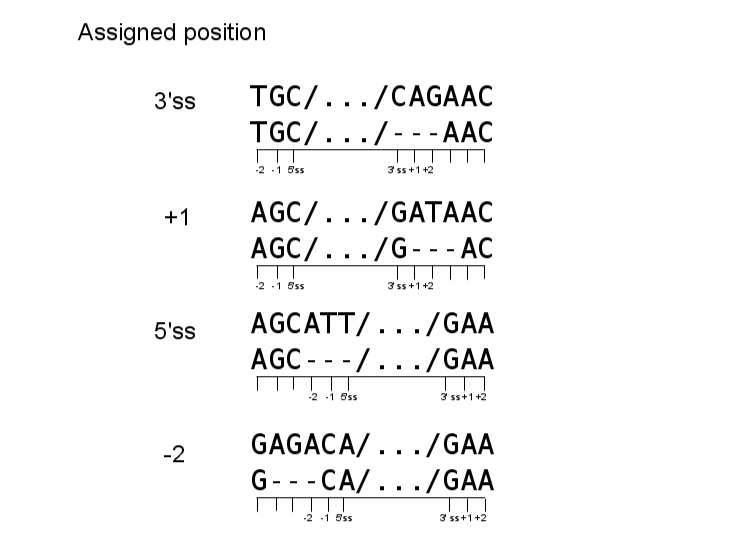

Supplement: Figure S9 — Numbering of alignment gaps relative to the 5′ and 3′ splice sites. Examples shown in the figure illustrate the numbering system used for assessing gap positions relative to the 5′ and 3′ splice sites. The splice sites are numbered 0, and gap position is numbered relative to the nearest splice site. Gaps that could not be unambiguously assigned to one splice site were very rare and their inclusion or exclusion did not affect our conclusions. (TIFF) [file pbio.1001229.s009.tiff]

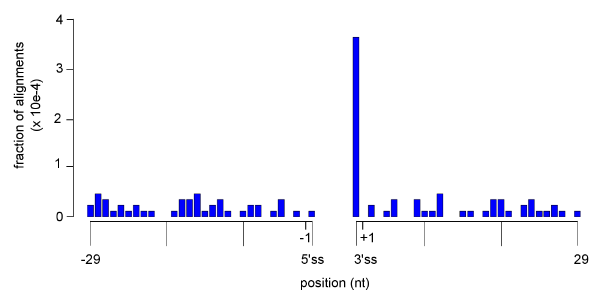

Supplement: Figure S10 — Exons with constitutively spliced NAGNAGs show an enrichment for gaps at the 3′ splice site. We restricted our analysis in Figure 5B to exons containing NAGNAGs which were constitutively spliced (ψ<5% or ψ>95% across all tissues) in both human and mouse. We observed qualitatively similar patterns of specific enrichment of gaps at the 3′ splice site, suggesting that the signal observed in Figure 5B was not due to unannotated alternative splicing of NAGNAGs. (TIFF) [file pbio.1001229.s010.tiff]

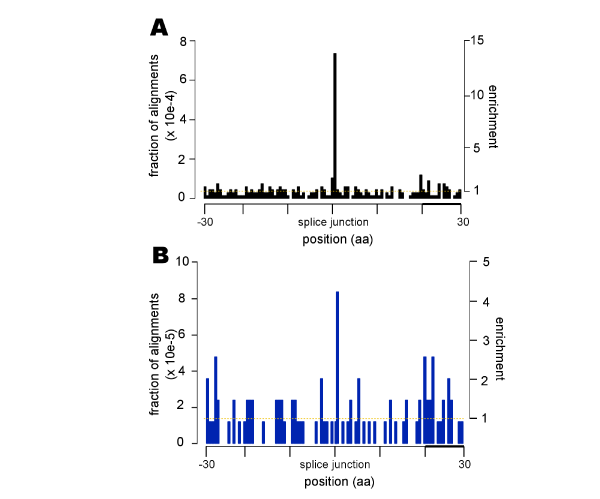

Supplement: Figure S11 — Alignment gaps at splice sites are enriched for predicted phosphorylation sites. The distribution of alignment gaps containing one or more predicted phosphorylation sites is shown for (A) all gaps and (B) gaps of three bases. (TIFF) [file pbio.1001229.s011.tiff]
