# Supplementary material for: Alternative Splicing of RNA Triplets Is Often Regulated and Accelerates Proteome Evolution
Source: PLoS Biol. 2012 Jan 3;10(1):e1001229. doi: 10.1371/journal.pbio.1001229 (PMC3250501; doi:10.1371/journal.pbio.1001229)
Supplement: Table S1 — Abundance and regulation of alternative splicing events in human protein-coding sequence (paired-end sequencing). “No. of events” shows the abundance of alternative splicing events in protein-coding sequence, restricted to events for which (1) neither isoform is predicted to be targeted by nonsense-mediated decay (no splice junction ≥50 nt downstream of the stop codon), and (2) both isoforms are expressed at ≥5% in at least one tissue. Isoform ratios are based on the numbers of reads aligning to the 3′ splice sites of each isoform, thereby treating each event as a choice between competing 3′ splice sites (with the exception of alternative 5′ splice site events, where the reads aligning to 5′ splice sites were used). This method ensures that the different classes of splicing events are analyzed “fairly,” irrespective of the length of the alternatively spliced sequence. “Fraction strongly regulated” gives raw estimates (not corrected for using FDRs based on technical replicates). (DOCX) [file pbio.1001229.s012.docx]

**Supplementary Table S1.** Abundance and regulation of alternative splicing events in human protein-coding sequence (paired-end sequencing).

|  | **No. of events^1^** | **No. of genes** | **Fraction regulated^2^** | **Fraction strongly regulated^3^** |
| --- | --- | --- | --- | --- |
| **skipped exon** | 6,276 | 4,364 | 84.5% | 52.9% |
| **NAGNAG** | 2,154 | 1,799 | 72.7% | 41.6% |
| **alternative 3' splice sites >3 nt apart** | 1,162 | 1,073 | 78.3% | 42.7% |
| **alternative 5' splice sites** | 893 | 835 | 79.5% | 45.4% |
| **mutually exclusive exons** | 128 | 124 | 84.4% | 54.7% |

**^1^**The abundance of alternative splicing events in protein coding sequence is shown, restricted to events for which: (1) neither isoform is predicted to be targeted by nonsense-mediated decay (no splice junction ≥ 50 nt downstream of the stop codon); and (2) both isoforms are expressed at ≥ 5% in at least one tissue.

**^2^**Isoform ratios are based on the numbers of reads aligning to the 3' splice sites of each isoform, thereby treating each event as a choice between competing 3' splice sites (with the exception of alternative 5' splice site events, where the reads aligning to 5' splice sites were used). This method ensures that the different classes of splicing events are analyzed “fairly,” irrespective of the length of the alternatively spliced sequence.

**^3^**These are “raw” estimates (not corrected for using FDRs based on technical replicates).
