# Supplementary material for: Alternative Splicing of RNA Triplets Is Often Regulated and Accelerates Proteome Evolution
Source: PLoS Biol. 2012 Jan 3;10(1):e1001229. doi: 10.1371/journal.pbio.1001229 (PMC3250501; doi:10.1371/journal.pbio.1001229)
Supplement: Table S3 — Abundance and regulation of alternative splicing events in human protein-coding sequence (single-end sequencing). Identical to Table S1, but based on single-end, rather than paired-end, sequence data from Body Map 2.0. (DOCX) [file pbio.1001229.s014.docx]

**Supplementary Table S3.** Abundance and regulation of alternative splicing events in human protein-coding sequence (single-end sequencing).

|  | **events** | **genes** | **regulated^1^** | **strongly**  **regulated^1^** |
| --- | --- | --- | --- | --- |
| **skipped exon** | 6,066 | 4,193 | 81.9% | 51.4% |
| **NAGNAG** | 2,028 | 1,717 | 67.8% | 38.1% |
| **alternative 3' splice sites**  **(>3 nt apart)** | 1,100 | 1,019 | 74.1% | 40.4% |
| **alternative 5' splice sites** | 849 | 807 | 76.1% | 45.0% |
| **mutually exclusive exons** | 112 | 109 | 87.5% | 60.7% |

**^1^**Identical in format to Supplementary Table S1, but based on single-end rather than paired-end sequence data from Body Map 2.0.
