# Supplementary material for: Alternative Splicing of RNA Triplets Is Often Regulated and Accelerates Proteome Evolution
Source: PLoS Biol. 2012 Jan 3;10(1):e1001229. doi: 10.1371/journal.pbio.1001229 (PMC3250501; doi:10.1371/journal.pbio.1001229)
Supplement: Table S4 — Abundance and regulation of alternative splicing events in mouse protein-coding sequence. Similar to Table S1, but for mouse NAGNAGs and based on paired-end sequencing of a C57BL/6J individual. (DOCX) [file pbio.1001229.s015.docx]

**Supplementary Table S4.** Abundance and regulation of alternative splicing events in mouse protein-coding sequence.

|  | **No. of events^1^** | **No. of genes** | **Fraction regulated** | **Fraction strongly regulated** |
| --- | --- | --- | --- | --- |
| **skipped exon** | 2,165 | 1,684 | 76.7% | 55.0% |
| **NAGNAG** | 1,970 | 1,658 | 28.3% | 8.38% |
| **alternative 3' splice sites >3 nt apart** | 443 | 421 | 49.7% | 26.0% |
| **alternative 5' splice sites** | 358 | 348 | 50.6% | 27.1% |
| **mutually exclusive exons** | 73 | 72 | 83.6% | 68.5% |

**^1^**Similar to Supplementary Table S1, but for mouse NAGNAGs and based on paired-end sequencing of tissues of a C57BL/6J individual.
