# Supplementary material for: Alternative Splicing of RNA Triplets Is Often Regulated and Accelerates Proteome Evolution
Source: PLoS Biol. 2012 Jan 3;10(1):e1001229. doi: 10.1371/journal.pbio.1001229 (PMC3250501; doi:10.1371/journal.pbio.1001229)
Supplement: Table S6 — Models for evolutionary gain and loss of single codons at 3′ splice sites resulting from splicing changes caused by single nucleotide substitutions. For each major category of change (bold headings in first column), the second column diagrams the effect on splicing, e.g., /… ⇒ …/ indicates a three base shift downstream in the location of the 3′ splice site, and …/ ⇒ /…/ indicates a change from constitutive splicing to alternative splicing at both the original 3′ splice site and a site three bases upstream. Below each diagram is a sequence motif consisting of specific bases (A, G), degenerate positions (N, indicating any base), or partially degenerate positions (B = “not A” = a C, G or T, H = “not G”, etc.). The third column lists conditions that are expected to favor each type of change (based on −3 base preferences shown in Figure 3B). (DOCX) [file pbio.1001229.s017.docx]

**Supplementary Table S6.** Models for evolutionary gain and loss of single codons at 3' splice sites resulting from splicing changes caused by single nucleotide substitutions.

| **Description** | **Pattern of splicing/sequence^1^** | **Required/favorable conditions^2^** |
| --- | --- | --- |
| **Exon contraction** | **/... ⇒ .../** |  |
| Upstream NAG creation | N_1_BGN_2_AG/ ⇒ N_1_AG/N_2_AG | N_1_ > N_2_ as -3 base |
| Upstream NAG creation | N_1_AHN_2_AG/ ⇒ N_1_AG/N_2_AG | N_1_ > N_2_ as -3 base |
|  |  |  |
| **Exon expansion** | **.../ ⇒ /...** |  |
| Cryptic NAG activation | N_1_AG/N_2_AG ⇒ N_1_BGN_2_AG/ | N_1_ > N_2_ as -3 base |
|  | N_1_AG/N_2_AG ⇒ N_1_AHN_2_AG/ | N_1_ > N_2_ as -3 base |
| -3 base preference reversal | N_1_AG/N_2_AG ⇒ N_1_’AGN_2_AG/ | N_1_ > N_2_ > N_1_’ as -3 base |
|  |  |  |
| **Partial contraction** | **.../ ⇒ /.../** |  |
| Upstream NAG creation | N_1_BGN_2_AG/ ⇒ N_1_AG/N_2_AG/ | N_1_,N_2_ not too different as -3 base |
| Upstream NAG creation | N_1_AHN_2_AG/ ⇒ N_1_AG/N_2_AG/ | N_1_,N_2_ not too different as -3 base |
| Upstream -3 base strengthening | N_1_AGN_2_AG/ ⇒ N_1_’AG/N_2_AG/ | N_1_ < N_2_ ≈ N_1_’ as -3 base |
|  |  |  |
| **Partial expansion** | **/... ⇒ /.../** |  |
| Downstream NAG creation | N_1_AG/N_2_BG ⇒ N_1_AG/N_2_AG/ | N_1_,N_2_ not too different as -3 base |
| Downstream NAG creation | N_1_AG/N_2_AH ⇒ N_1_AG/N_2_AG/ | N_1_,N_2_ not too different as -3 base |
| Upstream -3 base weakening | N_1_AG/N_2_AG ⇒ N_1_’AG/N_2_AG/ | N_2_ ≈ N_1_’ < N_1_ as -3 base |
|  |  |  |
| **Alternative expansion** | **/.../ ⇒ /...** |  |
| Downstream NAG loss | N_1_AG/N_2_AG/ ⇒ N_1_AG/N_2_BG | N_1_,N_2_ not too different as -3 base |
| Downstream NAG loss | N_1_AG/N_2_AG/ ⇒ N_1_AG/N_2_AH | N_1_,N_2_ not too different as -3 base |
| **Alternative contraction** | **/.../ ⇒ .../** |  |
| Upstream NAG loss | N_1_AG/N_2_AG/ ⇒ N_1_BGN_2_AG/ | N_1_,N_2_ not too different as -3 base |
| Upstream NAG loss | N_1_AG/N_2_AG/ ⇒ N_1_AHN_2_AG/ | N_1_,N_2_ not too different as -3 base |

^1^For each major category of change (bold headings in first column), the second column diagrams the effect on splicing, e.g., /... **⇒** .../ indicates a 3 base shift downstream in the location of the 3' splice site, .../ **⇒** /.../ indicates a change from constitutive splicing to alternative splicing at both the original 3' splice site and a site 3 bases upstream. Below each diagram is a sequence motif consisting of specific bases (A, G), degenerate positions (N, indicating any base), or partially degenerate positions (B = “not A” = a C, G or T, H = “not G”, etc.).

**^2^**The third column lists conditions that are expected to favor each type of change (based on -3 base preferences shown in Fig. 3B).
