# Supplementary material for: Alternative Splicing of RNA Triplets Is Often Regulated and Accelerates Proteome Evolution
Source: PLoS Biol. 2012 Jan 3;10(1):e1001229. doi: 10.1371/journal.pbio.1001229 (PMC3250501; doi:10.1371/journal.pbio.1001229)
Supplement: Table S7 — Number of amino acids gained or lost through 3′ splice site gaps are strongly biased depending on the intron phase. Table shows counts of amino acids gained/lost between orthologous human and mouse exons; the corresponding frequencies are shown in Figure 4I. (DOCX) [file pbio.1001229.s018.docx]

**Supplementary Table S7.** Number of amino acids gained or lost through 3' splice site gaps are strongly biased depending on the intron phase. Table shows counts of amino acids gained/lost between orthologous human and mouse exons; the corresponding frequencies are shown in Fig. 5I.
